# A zinc finger protein BBX19 interacts with ABF3 to affect drought tolerance negatively in chrysanthemum

**DOI:** 10.1111/tpj.14863

**Published:** 2020-07-21

**Authors:** Yanjie Xu, Xin Zhao, Palinuer Aiwaili, Xianying Mu, Meng Zhao, Jian Zhao, Lina Cheng, Chao Ma, Junping Gao, Bo Hong

**Affiliations:** ^1^ State Key Laborary of Agrobiotechnology Beijing Key Laboratory of Development and Quality Control of Ornamental Crops Department of Ornamental Horticulture College of Horticulture China Agricultural University Beijing 100193 China

**Keywords:** drought stress, hormone signaling, transcription factors, CmBBX19, CmABF3, abscisic acid, *Chrysanthemum morifolium*

## Abstract

Drought is an environmental factor that can severely influence plant development and distribution, and greatly affect the yield and economic value of crops. We characterized *CmBBX19*, a BBX family subgroup IV member gene, from the transcriptome database of *Chrysanthemum morifolium* in response to drought stress. Drought stress and ABA treatments downregulated the expression of *CmBBX19*. We generated *CmBBX19*‐overexpressing (*CmBBX19*‐OX) lines and *CmBBX19*‐suppressing lines (*CmBBX19*‐RNAi), and found that suppressed expression of *CmBBX19* led to enhanced drought tolerance compared with the wild‐type (WT) controls, while *CmBBX19*‐OX lines exhibited reduced drought tolerance. Downstream gene analysis showed that *CmBBX19* modulates drought tolerance mainly through inducing changes in the expression of ABA‐dependent pathway genes, including protective protein, redox balance and cell wall biogenesis genes, such as *responsive to ABA 18*, *peroxidase 12*, and *cellulose synthase‐like protein G2*. Moreover, CmBBX19 was shown to interact with CmABF3, a master ABA signaling component, to suppress expression of these downstream genes. We conclude that BBX19‐ABF3 module functions in the regulation of drought tolerance of chrysanthemum through an ABA‐dependent pathway.

## Significance Statement

Our work found that, (i) CmBBX19 acts as an EAR repressor during the drought stress response, and (ii) BBX19‐ABF3 module functions in the regulation of drought tolerance of chrysanthemum through an ABA‐dependent pathway.

## INTRODUCTION

Drought stress is a global phenomenon that seriously limits crop production and distribution. Given that plants are mostly sessile, and thus unable to relocate to areas with more water, they have evolved complex morphological, physiological, cellular and molecular level systems to cope with drought stress (Yamaguchi‐Shinozaki and Shinozaki, 2006; Yoshida *et al*., 2014). One of these involves the phytohormone abscisic acid (ABA), which plays a key role in several biological processes, such as plant growth and development, and in adaptive responses to environmental stress. It has been well known that there are two separate, but well‐connected, pathways mediating drought stress responses, i.e. the ABA‐dependent and independent drought pathways (Yamaguchi‐Shinozaki and Shinozaki, 2006; Fujita *et al*., 2011; Yoshida *et al*., 2014).

ABA signaling involves three essential core components: (i) ABA receptors known as the pyrabactin resistance (PYR)/pyrabactin resistance‐like (PYL)/regulatory component of the ABA receptor (RCAR) proteins; (ii) protein phosphatase 2C (PP2C) proteins; and (iii) sucrose‐non‐fermenting 1‐related protein kinase (SnRK) proteins (Cutler *et al*., 2010; Raghavendra *et al*., 2010). In the presence of ABA, PYR1/PYL/RCAR proteins directly inhibit the activity of PP2C proteins, leading to the derepression of SnRK2 isoforms (Raghavendra *et al*., 2010). These activated SnRK2 proteins then phosphorylate downstream transcription factors, which further trigger the transcription of ABA‐responsive genes, such as *RAB18*, *RD29B* and *ADH1* (Yoshida *et al*., 2014).

In studies with *Arabidopsis thaliana*, among the transcription factors that are phosphorylated by SnRK2 proteins during ABA signaling, three have been highlighted for their key involvement in drought stress responses, i.e. ABF2/AREB1, ABF3 and ABF4/AREB2 (Yoshida *et al*., 2015). These transcription factors activate the transcription of their downstream target genes by binding to an ABA‐responsive element (ABRE, PyACGTGG/TG) in the promoter (Yamaguchi‐Shinozaki and Shinozaki, 2006; Yoshida *et al*., 2014). It has been well‐documented that ABF/AREB proteins are basic leucine zipper (bZIP) transcription factors that can form hetero‐ or homodimers in the nucleus (Yoshida *et al*., 2010). However, little is known about their possible interactions with other transcription factors or associated mechanisms related to abiotic stress tolerance.

Another class of proteins that function in abiotic stress tolerance are members of the B‐box (BBX) family, a subgroup of the zinc finger proteins with one or two B‐box domains in their N terminus (Klug and Schwabe, 1995; Gangappa and Botto, 2014). The B‐box domain is responsible for transcriptional regulation and protein interaction (Khanna *et al*., 2009; Gangappa and Botto, 2014), and BBX family members can be classified into five subgroups based on the number of B‐box and CCT domains. BBX proteins also function in light‐regulated developmental processes, such as seedling photomorphogenesis, shade avoidance and photoperiod‐regulated flowering (Khanna *et al*., 2009; Gangappa and Botto, 2014; Vaishak *et al*., 2019). BBX18 represses thermotolerance in *A. thaliana* (Wang *et al*., 2013), while BBX24 induces salinity tolerance (Nagaoka and Takano, 2003). Heterologous overexpression of a chrysanthemum (*Chrysanthemum morifolium*) *BBX* gene, *CmBBX22*, in *A. thaliana* was shown to improve drought tolerance and delay leaf senescence (Liu *et al*., 2019), and we previously reported that *CmBBX24* has dual roles in modulating abiotic stress and flowering time (Yang *et al*., 2014).

Several studies have investigated the mechanisms by which BBX proteins affect abiotic stress responses. *Arabidopsis thaliana* BBX18 modulates the expression of a group of heat shock‐responsive genes (Wang *et al*., 2013), while CmBBX24 modulates gibberellin biosynthesis in chrysanthemum (Yang *et al*., 2014). Moreover, when overexpressed in *A. thaliana,* CmBBX22 functions in drought tolerance through transcriptional activation of downstream genes in the ABA signaling pathway, such as *ABI3* and *ABI5* (Liu *et al*., 2019), suggesting that BBX proteins may mediate ABA signaling. Another example of a study suggesting a relationship between BBX proteins and components of ABA signaling was the observation that BBX21 acts as a suppressor of the ABA control of seed germination through its physical interaction with ABI5 and via binding to the *ABI5* promoter (Xu *et al*., 2014; Kang *et al*., 2018). Recently, it was also shown that BBX19 acts as a repressor of seed germination by directly regulating *ABI5* expression (Bai *et al*., 2019). However, it has not been determined whether an interaction exists between BBX proteins and components of ABA signaling during responses to abiotic stress, including drought stress.

In present work, we tested the hypothesis that BBX proteins function in drought stress responses through interaction with other transcription factors, and in association with ABA signaling, using the commercially important ornamental plant chrysanthemum as an experimental system. Chrysanthemum is grown worldwide, but drought severely limits its planting areas and productivity, and so elucidating the molecular regulatory mechanisms of drought tolerance has considerable potential valuable for developing water‐efficient germplasm.

## RESULTS

### *CmBBX19* expression is downregulated by drought

We identified four unigenes encoding putative BBX group IV subfamily proteins in a chrysanthemum dehydration response transcriptome database (Xu *et al*., 2013). Of these, UN68402, was downregulated by dehydration treatment (Table S1). Sequence alignment and phylogenetic analysis revealed that the predicted protein contains structural characteristics of BBX group IV from *A. thaliana* and that it is a homolog of AtBBX18 and AtBBX19 (Figure S1). Accordingly, we named the gene *CmBBX19*, following the BBX family protein nomenclature system (Khanna *et al*., 2009).

**Figure 1:**
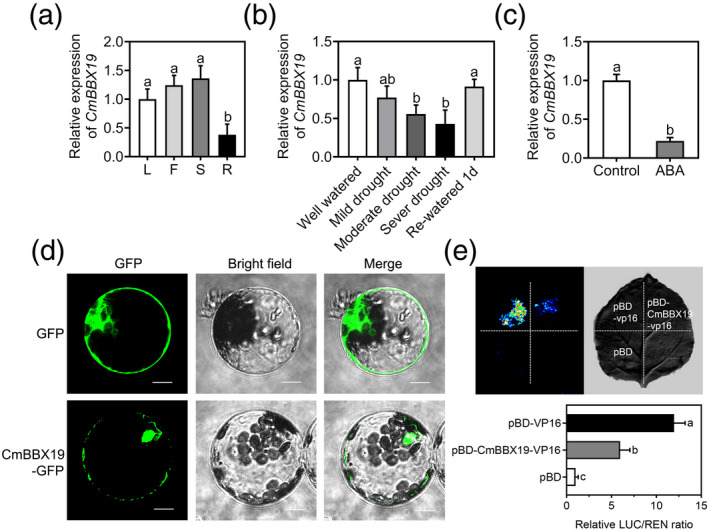
Expression patterns, subcellular localization and transactivation of CmBBX19. (a) *CmBBX19* transcript abundance in different organs, based on quantitative real‐time polymerase chain reaction analysis. L, leaves; F, flowers; S, stems and R, roots. Samples were collected at Zeitgeber time 3 from dawn (ZT3). (b) *CmBBX19* transcript abundance in leaves over a drought time course. Relative water contents of the soil for the indicated treatments were 77% (well‐watered), 56% (mild drought), 25% (moderate drought), 11% (severe drought) and 77% (1 day rewatered). Leaves were harvested after each treatment at ZT3. (c) *CmBBX19* transcript abundance following 100 μm abscisic acid (ABA) treatment. Samples were harvested after 1 h of ABA treatment. *CmUBIQUITIN* was used as the internal control. (d) Subcellular localization of the CmBBX19‐GFP fusion protein in chrysanthemum protoplasts was observed by confocal laser scanning microscopy. GFP, green fluorescent protein; CmBBX19‐GFP, CmBBX19‐GFP fusion protein. Images are dark field (left) showing green fluorescence, bright field (middle) showing the morphology of the cells, and merged (right) showing a combination. Scale bars: 10 µm. (e) Transactivation activity analysis of the CmBBX19 protein in *Nicotiana benthamiana* leaves. Plasmid combinations of the dual LUC/REN reporter and effectors consisting of pBD‐CmBBX19‐VP16, pBD‐VP16 or pBD were co‐transformed into *N. benthamiana* leaves. LUC and REN activities were assayed 3 days after infiltration. Representative photographs show firefly luciferase fluorescence signals when the corresponding effectors were introduced into *N. benthamiana* leaves. Three independent experiments were performed and error bars indicate standard deviations. Significant differences were determined by Duncan's multiple range test (*P* < 0.05).

*CmBBX19* expression was evaluated in different organs and relatively high transcript abundance was found in leaves, flowers and stems, but lower levels in roots (Figure 1a). To test the response of *CmBBX19* to drought stress, we used quantitative real‐time PCR to measure its expression in mature leaves from soil‐grown chrysanthemum plants taken over a drought time course. *CmBBX19* expression decreased following the application of drought stress, and following severe drought stress (about 11% relative water content of soil), expression levels were 45% of those in well‐watered control plants, but then returned to basal levels 1 day after rewatering (Figure 1b).

As endogenous ABA levels tend to rise rapidly in response to drought stress, we next tested whether ABA could affect *CmBBX19* transcription. As shown in Figure 1(c), *CmBBX19* expression decreased to 0.2‐fold after 1 h of exogenous ABA treatment, suggesting that CmBBX19 may act as a component of the ABA signaling pathway under drought stress.

To confirm whether CmBBX19 functions as a transcription factor, we transiently expressed a CmBBX19‐green fluorescent protein (GFP) fusion protein in chrysanthemum protoplasts. The fusion protein was mainly located in the nucleus, but was also detected in the cytoplasm, while the GFP protein alone was present throughout the cell (Figure 1d).

As CmBBX19 contains a typical ethylene‐responsive element binding factor‐associated amphiphilic repression (EAR) motif in its C terminus, it can be considered a transcriptional repressor (Wang *et al*., 2014). Based on our sequence analysis of the BBX family in chrysanthemum, among the more than 18 BBX family members only CmBBX19 has an EAR motif. To determine whether CmBBX19 has transcriptional repression activity, we performed a dual‐luciferase transactivation assay. As shown in Figure 1(e), leaves of *Nicotiana benthamiana* expressing the CmBBX19‐VP16 construct exhibited much lower relative luciferase activity compared with the control harboring VP16 alone, indicating that CmBBX19 is a transcriptional repressor.

### CmBBX19 affects drought stress tolerance

**Figure 2:**
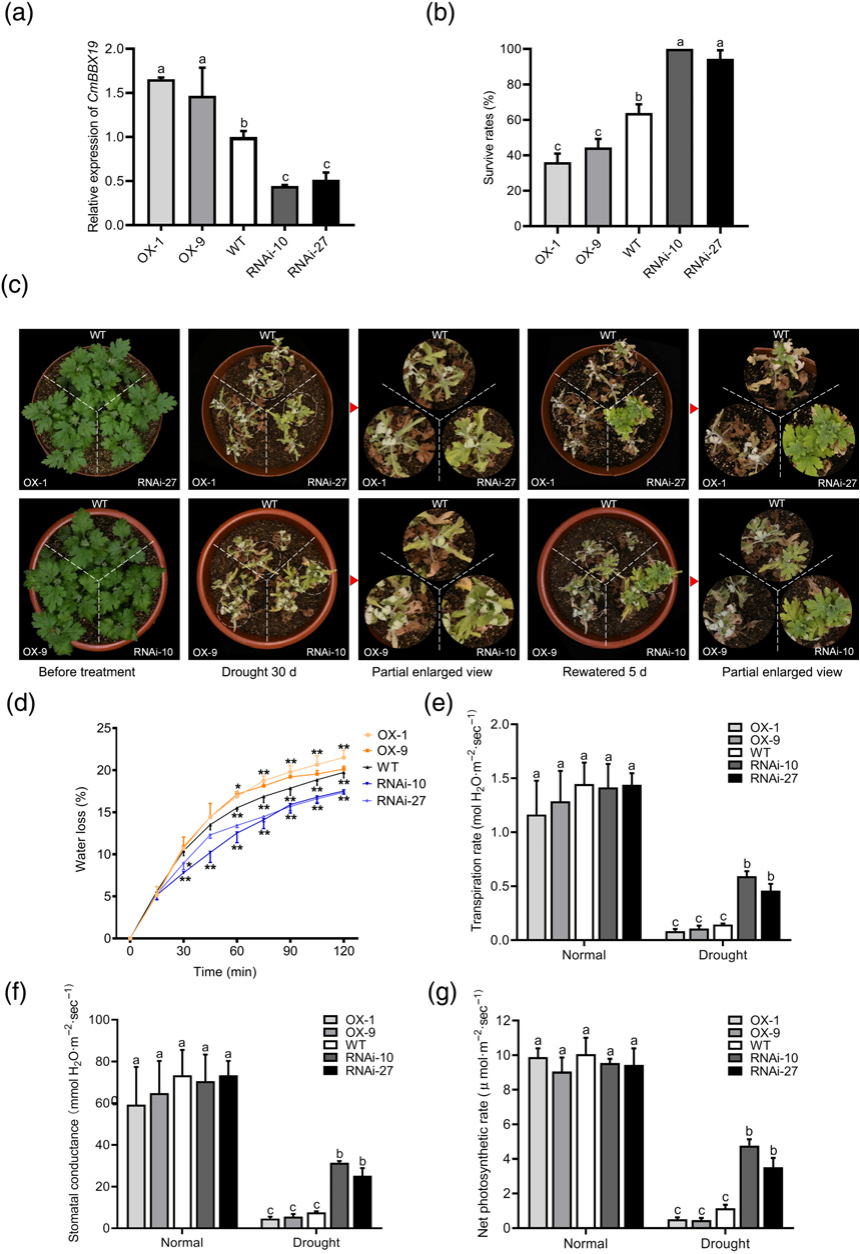
Drought stress tolerance of *CmBBX19*‐overexpressing (*CmBBX19*‐OX) or *CmBBX19*‐suppressed (*CmBBX19*‐RNAi) chrysanthemum plants. (a) Expression of *CmBBX19* in wild‐type (WT) and transgenic plants at Zeitgeber time 3 from dawn (ZT3), determined by quantitative real‐time polymerase chain reaction. *CmBBX19*‐OX‐1 and ‐9 correspond to two independent *CmBBX19*‐OX lines, and *CmBBX19*‐RNAi‐10 and ‐27 correspond to two independent *CmBBX19*‐RNAi lines. (b) Phenotypes of *CmBBX19*‐OX or *CmBBX19*‐RNAi plants under drought stress conditions compared with WT plants. Four‐week‐old *CmBBX19*‐OX, *CmBBX19*‐RNAi and WT plants were planted in one pot and water was withheld for 30 days, before rewatering and recovery for 5 days with regular watering. (c) Survival rates of *CmBBX19*‐OX, *CmBBX19*‐RNAi and WT plants grown under drought stress conditions. Three independent experiments were performed, for each experiment, 24 WT plants, 12 *CmBBX19*‐OX plants and 12 *CmBBX19*‐RNAi plants were used (*n* = 12), error bars indicate standard deviation (SD). Significant differences were determined by Duncan's multiple range test (*P* < 0.05). (d) Water loss in leaves of *CmBBX19* transgenic lines and WT plants. Water loss assays were performed for 120 min (*n* = 5). Asterisks indicate signiﬁcant differences compared with WT as determined by Tukey’s honestly signiﬁcant difference method (*P < 0.05; **P < 0.01). (e–g) Transpiration rate (e), stomatal conductance (f), and photosynthetic rate (g) of the *CmBBX19* transgenic lines and WT under normal and drought stress conditions (*n* > 5).

To determine whether CmBBX19 functions in the regulation of drought stress tolerance, we created 19 *CmBBX19*‐overexpressing chrysanthemum lines (*CmBBX19*‐OX) and 30 *CmBBX19*‐suppressed (RNA interference, *CmBBX19*‐RNAi) lines. We chose two *CmBBX19*‐OX and two *CmBBX19*‐RNAi lines to compare with wild‐type (WT) plants. The transcript abundance of *CmBBX19* in the transgenic lines was confirmed by quantitative real‐time PCR analysis (Figure 2a). We also determined the expression of other members of chrysanthemum BBX group IV in the *CmBBX19*‐RNAi lines and confirmed that only *CmBBX19* was silenced (Figure S2).

We also detected the protein levels of CmBBX19‐GFP in *CmBBX19*‐OX lines by western blotting analysis. The results showed that substantially higher protein levels were observed in CmBBX19‐OX than WT plants (Figure S3b), further confirming the genetic transformation of *CmBBX19* at post‐transcription level.

To examine the effect of *CmBBX19* expression on the tolerance of drought stress, we grew the overexpressing and silenced lines in soil under normal watering conditions, then after 7 days, the plants were treated by withholding water for 30 days, followed by a 5‐day recovery process, and the survival rates were determined. Before the drought treatment, compared with the WT, the transgenic lines did not exhibit clear differences in growth. However, after a 30‐day drought treatment, the *CmBBX19*‐OX plants and WT plants showed drought‐induced damage that was more severe, such as wilted and withered leaves, than did the *CmBBX19‐*RNAi plants (Figure 2c). After a 5‐day recovery period, 67% of the WT and 100% of the *CmBBX19‐*RNAi plants survived and showed continued growth of their apical shoots, whereas the survival rate of the *CmBBX19*‐OX plants was 33%, and the surviving plants mostly and subsequently exhibited weak outgrowth of the lateral or basal shoots (Figure 2b).

To understand the physiological mechanisms of drought tolerance that were influenced by CmBBX19, we compared the transpiration rates, stomatal conductance, water loss and photosynthesis rates in transgenic plants and WT plants under drought/dehydration conditions. The transpiration rate, stomatal conductance levels, and photosynthesis rates were significantly higher in *CmBBX19‐*RNAi plants, whereas the water loss rate was significantly lower in *CmBBX19*‐RNAi plants than in the WT control under drought/dehydration conditions (Figure 2d–g). The *CmBBX19*‐OX plants exhibited the opposite effects. These results were consistent with CmBBX19 influencing multiple physiological processes that contribute to drought tolerance.

### CmBBX19 modulates the expression of abiotic stress‐responsive genes in the ABA‐dependent pathway

As described above, we observed that the exogenous ABA treatment downregulated *CmBBX19* expression (Figure 1c). Here, we tested whether ABA treatment affects the protein level of CmBBX19 in transgenic plants. The results showed that almost no difference of CmBBX19 protein was observed between ABA treatment and control in *CmBBX19*‐OX plants, indicating that the response of CmBBX19 to ABA mainly occurs at the transcription level, rather than post‐transcriptional level (Figure S3). Therefore, we investigated whether the action of CmBBX19 in response to drought stress was related to the ABA signaling pathway. Specifically, we carried out a large‐scale screen for differentially expressed genes between leaves from *CmBBX19*‐RNAi or *CmBBX19*‐OX plants and WT plants, using a RNA‐sequencing (RNA‐seq) approach.

**Figure 3:**
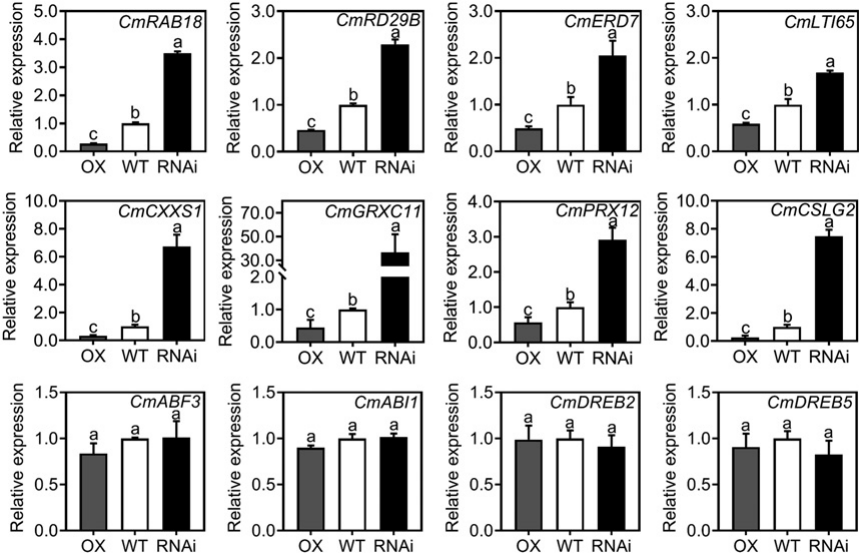
Expression of genes related to the abscisic acid (ABA)‐dependent or ‐independent pathway in *CmBBX19‐*OX and *CmBBX19*‐RNAi chrysanthemum plants. Quantitative real‐time polymerase chain reaction analysis was performed to evaluate the expression of each gene. *CmUBIQUITIN* was used as the control gene. Three independent experiments were performed and error bars indicate standard deviation. Letters indicate significant differences according to Duncan's multiple range test (*P* < 0.05).

We focused on the expression of genes related to the ABA‐dependent and ABA‐independent pathways in the transgenic plants compared with WT plants under normal growth conditions. ABA‐dependent pathway genes, such as *CmRAB18*, *CmRD29B*, *CmERD7* and *CmLTI65*, were downregulated in the *CmBBX19*‐OX plants but upregulated in *CmBBX19*‐RNAi plants, relative to WT. The expression of genes from the ABA‐independent pathway, including *CmDREB2* and *CmDREB5*, did not change in either *CmBBX19*‐OX or *CmBBX19*‐RNAi plants (Figure 3).

We then investigated whether the *CmBBX19*‐modulated drought tolerance was associated with changes in ABA biosynthesis by evaluating gene expression and measuring ABA levels in leaves. We did not observe significant differences in ABA content and expression of ABA biosynthesis‐related genes, such as *CmNCED* and *CmABA2* among the transgenic lines and WT (Figures S4 and S5). We also tested whether the effect of *CmBBX19* on drought tolerance was through transcriptional regulation of key ABA signaling components and downstream responsive genes. Among the transgenic lines and WT, we did not find a significant difference in the expression of the ABA receptor gene, *CmPYR*/*PYL*/*RCAR*, or core ABA signaling genes, such as *CmSnRK2*, *CmABI3*, *CmABI4*, *CmABI5* and *CmABF3* (Figure S5). However, we detected significant differences in the expression of genes involved in downstream ABA signaling that encoded late embryogenesis‐abundant (LEA)‐like protective proteins, such as CmRAB18 and CmRD29B; that encoded oxidation‐reduction proteins, such as peroxidase 12‐like (PRX12), glutaredoxin‐C11‐like (GRXC11), and C‐terminal cysteine residue is changed to a serine 1 (CXXS1); that encoded cell wall biogenesis‐related proteins, such as cellulose synthase‐like protein G2 (CmCSLG2). The expressions of these genes above were downregulated in the *CmBBX19*‐OX plants but upregulated in *CmBBX19*‐RNAi plants, relative to WT (Figure 3 and Table S2).

A recent study reported that overexpression in *A. thaliana* of *CmBBX22*, another chrysanthemum BBX gene family member, can improve drought tolerance through delaying leaf senescence (Liu *et al*., 2019). Here, we tested the expression of leaf senescence‐related genes, such as *CmNYC1* and *CmNYE1*, in transgenic and WT plants and showed that CmBBX19 did not affect the expression of leaf senescence‐related genes (Figure S5b). This indicates that CmBBX19 functions in drought tolerance through a different regulatory mechanism from that associated with CmBBX22.

Based on these findings, we concluded that CmBBX19 influences drought tolerance mainly through modulating the accumulation of protective proteins, maintaining cellular redox balance, and promoting cell wall biogenesis in the ABA‐dependent pathway, rather than by altering ABA biosynthesis and key ABA signaling components at the transcriptional level.

### CmBBX19 modulates its downstream genes through interacting with CmABF3

**Figure 4:**
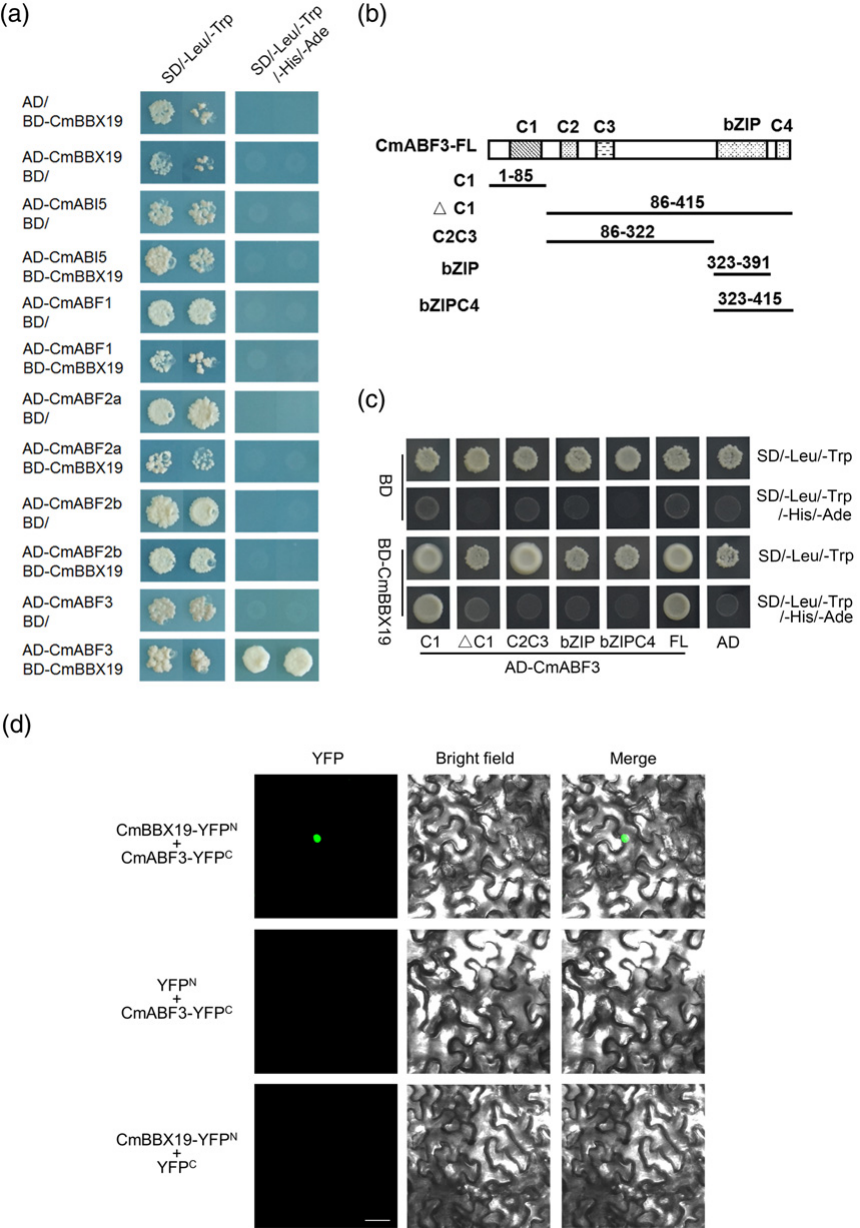
Interaction of CmBBX19 with the abscisic acid signaling component, CmABF3, using a yeast two‐hybrid assay and bimolecular fluorescence complementation. (a) Yeast two‐hybrid screening of the CmBBX19 and CmABF proteins. Diploid yeast cells containing both the bait BD‐CmBBX19 construct and the prey AD‐ABFs constructs were obtained by mating. Overnight cultures were normalized to an OD_600_ of 0.1 and spotted on to non‐selective medium lacking Leu and Trp (left panel) and selective medium lacking Leu, Trp, His and Ade (right panel). Negative controls contained empty bait and/or prey vectors. (b) Schematic representation of the conserved domains in CmABF3 and the truncated CmABF3 variants tested in the assays. (c) Yeast two‐hybrid assays evaluating the interaction between CmBBX19 and the truncated CmABF3. (d) Interaction of CmBBX19 and CmABF3 in a bimolecular fluorescence complementation assay. *Nicotiana benthamiana* leaves were co‐infiltrated with CmBBX19‐YFP^N^ and CmABF3‐YFP^C^ constructs and visualized by confocal microscopy 3 days after infiltration. Combinations of CmBBX19‐YFP^N^ and YFP^C^, and YFP^N^ and CmABF3‐YFP^C^ were used as negative controls. Scale bars, 40 μm.

To elucidate how CmBBX19 affects the expression of downstream genes, we analyzed the promoters of chrysanthemum genes related to protective protein. We found that ABRE *cis*‐elements were enriched in the promoters of LEA protein genes (Figure S6). However, the expression of ABRE‐binding factors, *CmABF*s/*AREB*s, was not affected in *CmBBX19* transgenic lines (Figure 3 and Figure S5), so we investigated the possibility that CmBBX19 affects key ABA signaling components at the post‐transcriptional level. We identified five *CmABF*/*AREB*‐Like genes (*CmABF1*, *CmABF2a*, *CmABF2b*, *CmABF3* and *CmABI5*) based on sequence similarity and phylogenetic analysis (Figure S7). We took CmBBX19 as bait in a yeast two‐hybrid analysis (BD‐CmBBX19) and fused the five AREB‐like proteins to the Gal4 activation domain. The results showed that compared with controls, yeast coexpressing CmBBX19 and CmABF3 grew normally on selection medium, indicating that CmBBX19 physically interacts with CmABF3, but not with the other investigated proteins (Figure 4a).

To verify the CmBBX19‐CmABF3 interaction *in vivo*, we carried out a bimolecular fluorescence complementation (BiFC) assay. We observed strong signals of yellow fluorescent protein (YFP) in *N. benthamiana* leaf cells transiently coexpressing CmBBX19‐YFP^N^ (CmBBX19 fused with the N terminus of YFP) and CmABF3‐YFP^C^ (CmABF3 fused with the C terminus of YFP) (Figure 4d). In contrast, we did not observe any detectable YFP signals in the negative controls, CmBBX19‐YFP^N^ and YFP^C^, YFP^N^ and CmABF3‐YFP^C^. These results indicate that CmBBX19 can interact with CmABF3 *in vivo*.

According to previous studies, ABF/AREB proteins contain four conserved regions (C1–C4) and a bZIP domain (Jakoby *et al*., 2002; Fujita *et al*., 2005; Zhao *et al*., 2019). To determine which conserved CmABF3 domain interacted with CmBBX19, we generated multiple truncated forms of CmABF3 (Figure 4b) and found that only C1 interacted with CmBBX19 in a yeast two‐hybrid assay (Figure 4c). Previous studies also showed that the C1 domain of AREB1 has transactivation activity (Fujita *et al*., 2005) and contains a conserved RXXS/T site, which can be phosphorylated by SnRK2 protein kinases (Uno *et al*., 2000; Furihata *et al*., 2006). The interaction between CmBBX19 and CmABF3 suggested that CmBBX19 might affect ABA signaling by suppressing the transactivation activity of CmABF3, rather than by interfering with the promoter‐binding activity of CmABF3.

We also isolated CmBBX19 and CmABF3 homologs from *A. thaliana* to test the conservation of the interaction between BBX19 and ABF3 in another species. Yeast two‐hybrid and BiFC analyses showed that neither CmBBX19 nor AtBBX19 interacted with AtABF3 (Figure S8), suggesting that the interaction between BBX19 and ABF3 in chrysanthemum is not conserved in *A. thaliana*.

**Figure 5:**
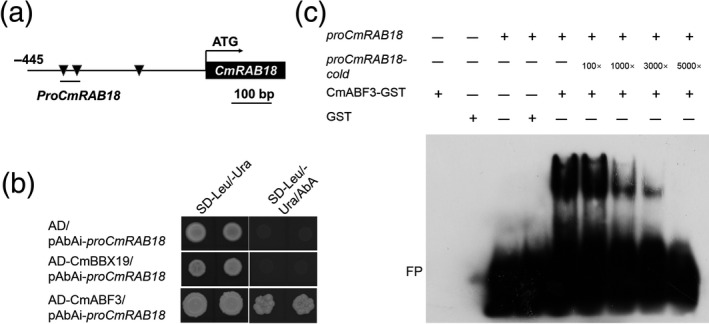
CmABF3 binds to the *CmRAB18* promoter independently of CmBBX19. (a) Schematic representation of the 445‐bp *CmRAB18* promoter. Triangles correspond to putative abscisic acid‐responsive element motifs. Line below the promoter indicates the fragment used in an electrophoretic mobility shift assay (−388/−339). (b) Analysis of CmABF3 and CmBBX19 binding to the *CmRAB18* promoter in a yeast one‐hybrid system. The empty prey vector (AD) was used as a negative control. Interactions between bait and prey were determined by cell growth on synthetic dropout nutrient medium lacking Leu and Ura, and containing 200 ng ml^−1^ Aureobasidin A. (c) Analysis of CmABF3 binding to the *CmRAB18* promoter using an electrophoretic mobility shift assay. Purified CmABF3 protein (3 μg) was incubated with 50 nm biotin‐labeled probes. For the competition test, cold probes at 100‐, 1000‐, 3000‐ or 5000‐fold concentrations were added to the experiment described above. FP, free probe.

**Figure 6:**
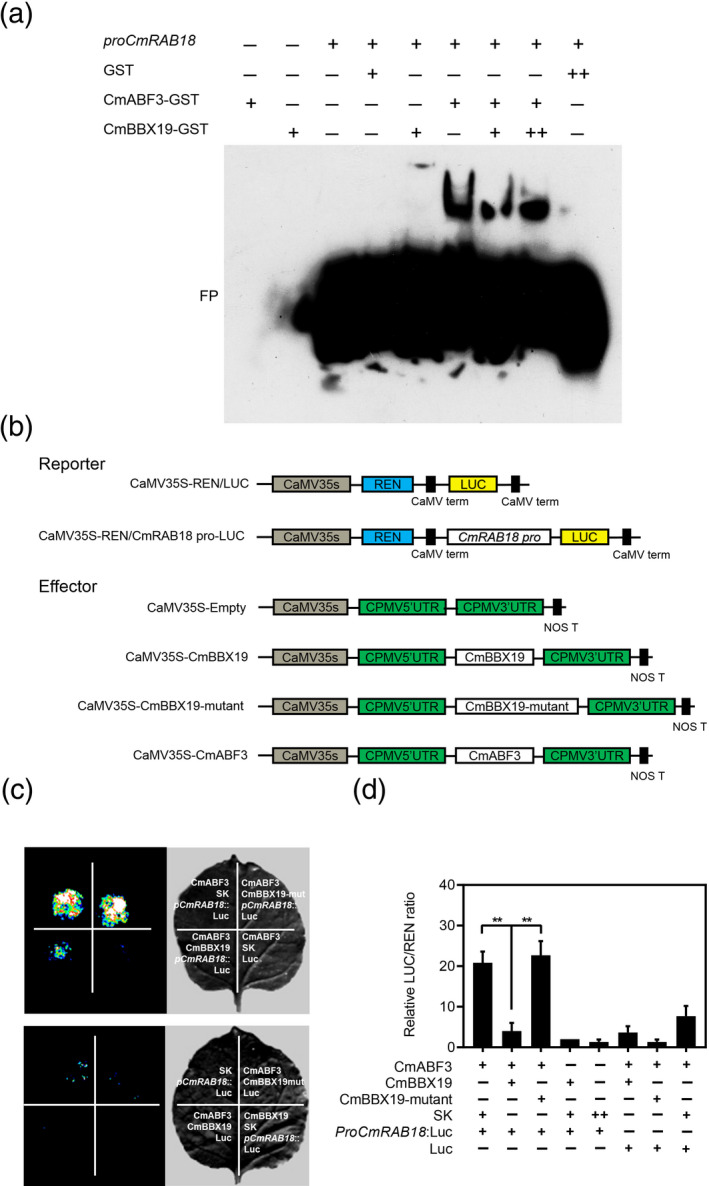
CmBBX19 interacts with CmABF3 to repress transcription of *CmRAB18*. (a) Analysis of CmABF3 and CmBBX19 binding to the *CmRAB18* promoter, based on an electrophoretic mobility shift assay. Purified protein (3 μg) was incubated with 50 nm biotin‐labeled probe. For the competition test, purified CmBBX19 protein, at 1‐ or 10‐fold concentrations, was added to the experiment described above. FP, free probe. (b) Schematic representation of the double‐reporter and effector plasmids used in the dual‐luciferase reporter assay. (c‐d) The interaction of CmABF3 or CmBBX19 with the *CmRAB18* promoter as shown by a dual luciferase (LUC) reporter system. A 445 bp *CmRAB18* promoter fragment was used. Constructs used in the assay are shown above. LUC vectors containing the *renilla luciferase* (*REN*) gene under the control of the 35S promoter were used as a positive control. Samples were infiltrated into *Nicotiana benthamiana* leaves, and LUC and REN activities were assayed 3 days after infiltration. Representative photographs are shown of firefly luciferase fluorescence signals (c) and relative LUC/REN ratio are shown of normalizing LUC activity to that of REN (d) when the corresponding effectors and reporters were introduced into *N. benthamiana* leaves. Three independent experiments were performed and error bars indicate standard deviations. Asterisks indicate signiﬁcant differences as determined by Tukey’s honestly significant difference method (**P < 0.01).

As BBX19 interacted with ABF3, we investigated whether BBX19 directly affects the expression of downstream genes. Sequence analysis showed that a 455 bp region of the *CmRAB18* promoter contains three ABRE motifs (Figure 5a), and in a yeast one‐hybrid assay CmABF3, but not CmBBX19, directly bound to the promoter of *CmRAB18* (Figure 5b). We used a 50 bp fragment of the *CmRAB18* promoter (position −388 to −339) that contains two ABRE motifs as a probe in an electrophoretic mobility shift assay (EMSA), and observed that the ABRE *cis*‐elements in the *CmRAB18* promoter were directly bound by CmABF3, but not by CmBBX19 (Figures 5c and 6a). We also performed EMSA to examine whether CmBBX19 interferes with the binding affinity of CmABF3 to the promoters of its target genes (Figure 6a). We saw that CmBBX19 had no effect on the binding affinity of CmABF3 to the *CmRAB18* promoter fragments, even after increasing the concentration of CmBBX19 in the reactions.

### CmBBX19 represses CmABF3 activation of *CmRAB18* expression

To determine whether CmBBX19 affects ABA‐responsive genes by suppressing the transactivation activity of ABF3, we performed a dual‐luciferase reporter assay. We fused the *CmRAB18* promoter to firefly luciferase (LUC) (*proCmRAB18*:LUC) to generate a reporter construct, and used 35S:CmABF3, 35S:CmBBX19 and a mutant BBX19 with a Cys‐25 to Ser substitution in B box1 (35S:CmBBX19mut) as three different effectors (Figure 6b). After co‐transforming 35S:CmABF3 and the *proCmRAB18*:LUC into *N. benthamiana* leaves, we observed significantly higher LUC activities than when the empty vector and *proCmRAB18*:LUC (SK+proCmRAB18) were co‐transformed. When we co‐transformed 35S:CmABF3, 35S:CmBBX19 and *proCmRAB18*:LUC (CmABF3 + CmBBX19 + pCmRAB18), significantly reduced LUC activity was observed (Figure 6c). Furthermore, when mutated CmBBX19 replaced CmBBX19, LUC activity was recovered.

**Figure 7:**
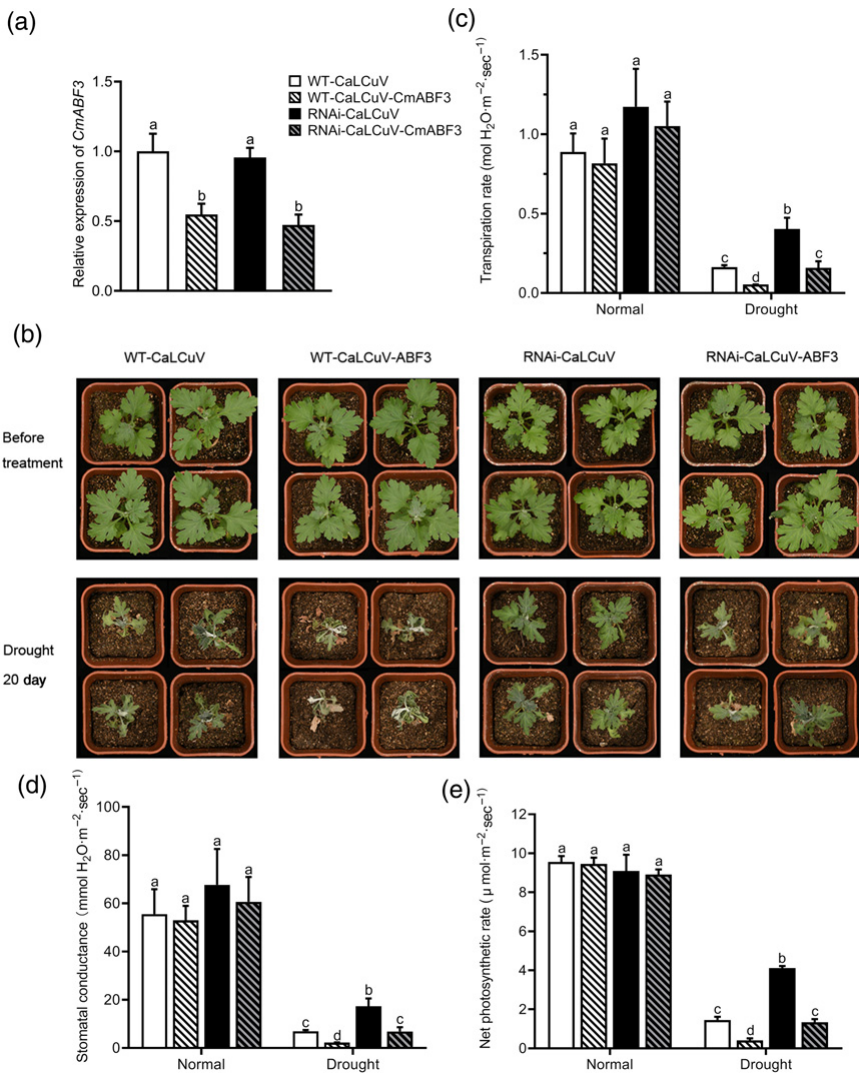
Drought stress tolerance of CaLCuV‐amiR‐ABF3‐infected wild‐type (WT) and *CmBBX19*‐RNAi chrysanthemum plants. (a) Expression of *CmABF3* in CaLCuV‐amiR‐ABF3‐infected WT and *CmBBX19*‐RNAi plants. (b) Phenotypes resulting from silencing of *CmABF3* in the WT and *CmBBX19*‐RNAi background after 20 days drought stress. (c–e) Transpiration rate (c), stomatal conductance (d) and photosynthetic rate (e) of WT and *CmBBX19*‐RNAi plants infected with CaLCuV or CaLCuV‐amiR‐ABF3 under normal and drought stress conditions (*n* > 5).

To obtain genetic evidence that CmBBX19 interacts with CmABF3, we silenced *CmABF3* in the WT and *CmBBX19*‐RNAi backgrounds, using a modified cabbage leaf‐curl geminivirus vector (CaLCuV) containing the artificial microRNA‐ABF3 (CaLCuV‐amiR‐ABF3) (Figure 7a). Silencing of *CmABF3* in the WT and *CmBBX19*‐RNAi backgrounds exacerbated the wilting symptoms after a 20‐day drought stress treatment, and the plants showed a reduced transpiration rate, stomatal conductance and net photosynthetic rate (Figure 7b–f). Silencing *CmABF3* significantly suppressed the *CmBBX19*‐RNAi‐induced upregulation of abiotic stress‐responsive genes, particularly the LEA protein genes *CmRAB18*, *CmRD29B*, *CmERD7* and *CmLTI65* (Figure S9). These results indicate that CmABF3 mediates CmBBX19‐influenced drought tolerance in chrysanthemum.

## DISCUSSION

### CmBBX19 attenuates drought tolerance in an ABA‐dependent manner

Continuous activation of plant stress responses is metabolically expensive when plants were grown under normal conditions, and runaway responses can cause self‐inflicted damage. Therefore, plants evolve a sophisticated system to control stress responses precisely (Kazan, 2006; Dong and Liu, 2010). Here, we found that a BBX family protein, CmBBX19, is a transcriptional repressor and functions as a negative regulator of drought tolerance in chrysanthemum. Under normal conditions, CmBBX19 suppresses the expression of a set of stress‐ and ABA‐responsive genes, such as *CmRAB18* and *CmRD29B*. Upon drought stress, expression of *CmBBX19* is downregulated to release those stress‐related genes to enhance drought tolerance of chrysanthemum.

CmBBX19 is the only member of the chrysanthemum BBX family containing an EAR motif. In plants, EAR motif‐containing proteins have been well‐documented as transcriptional repressors and function in a broad range of biological processes, including floral transition and meristem maintenance, as well as responses to hormones and both biotic and abiotic stresses (Kagale and Rozwadowski, 2011; Causier *et al*., 2012). Several EAR motif‐containing proteins were reported to affect plant drought response. For example, RELATED TO AP2 1 (RAP2.1), which functions as a negative “subregulon” of DREB‐type activators, takes part in the precise control of the expression of stress‐related genes in *A. thaliana* (Dong and Liu, 2010). In addition, it has been reported that a rice (*Oryza sativa*) drought‐responsive zinc finger protein, OsDRZ1, acts as a transcriptional repressor, and improves drought tolerance through enhancement of antioxidative protection and decrease of expression levels of drought‐responsive genes (Yuan *et al*., 2018). In particular, both RAP2.1 and OsDRZ1 modulate the drought response through a DREB‐dependent pathway, namely the ABA‐independent pathway. Besides, CmBBX19 influences the expression of genes related to ABA signaling, demonstrating that CmBBX19 functions in an ABA‐dependent manner, which is distinct to the previously reported drought‐responsive EAR‐containing proteins.

### CmBBX19 binds to CmABF3 and interferes with CmABF3‐activated expression of downstream genes

It has been reported that BBX21 can transcriptionally activate *ABI5* gene or interact with ABI5 protein to influence ABA signaling in *A. thaliana* (Xu *et al*., 2014; Kang *et al*., 2018).

Regarding BBX19, a previous report showed that BBX19 negatively modulate flowering time through interacting with CONSTANS (CO) to suppress FLOWERING LOCUS T (FT) transcription (Wang *et al*., 2014). Recently, BBX19 was found to activate expression of *ABI5* through directly binding to its promoter to suppress seed germination (Bai *et al*., 2019).

Interestingly, in the present work, we found that CmBBX19, a chrysanthemum homolog of BBX19, cannot bind to ABI5, but interacts with CmABF3, another key component of ABA signaling. In addition, our current data showed that BBX19 does not interact with ABF3 in *A. thaliana*, suggesting that the role of the CmBBX19‐CmABF3 module in drought tolerance might be chrysanthemum specific. However, similar to the regulatory manner of BBX19 in *A. thaliana*, CmBBX19 interfered with CmABF3‐dependent transactivation of downstream genes, instead of directly binding to the promoter of those genes.

**Figure 8:**
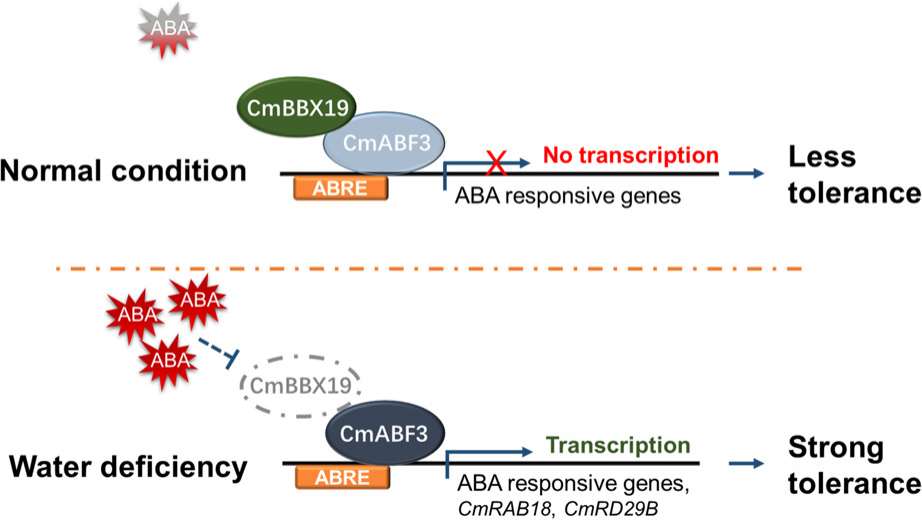
Schematic model of BBX19‐ABF3 interaction involved in the drought stress response in chrysanthemum. Under normal conditions, CmBBX19 binds to CmABF3 and suppresses the activation by CmABF3 of abscisic acid (ABA)‐responsive genes. Under drought stress conditions, accumulated ABA reduces the expression of *CmBBX19*, thereby relieving CmABF3 from suppression and activating expression of ABA‐responsive genes, such as *CmRAB18* and *CmRD29B*. ABRE, abscisic acid‐responsive element.

In conclusion, we suggest a working model (Figure 8) in which CmBBX19 functions as a transcriptional suppressor in the drought stress response through interaction with CmABF3, and suppresses CmABF3 activation of target genes, thereby tightly influencing drought stress responses.

## EXPERIMENTAL PROCEDURES

### Plant materials and treatments

We took a chrysanthemum cultivar (*Chrysanthemum morifolium*, cv. Fall Color) as plant material in present study. Plant cultivation was carried out as previously described (Wei *et al*., 2017). Forty‐day‐old tissue culture plantlets were transplanted into 9‐cm diameter pots filled with a peat/vermiculite (1:1, v/v) mixture and grown in a controlled environment (23 ± 1°C, 40% relative humidity, 100 μmol m^−2^ sec^−1^ illumination and 16 h light/8 h dark).

For the expression determination of *CmBBX19* in different chrysanthemum organs, plants were grown under long‐day (16 h light/8 h dark) conditions for 6 months.

For different degrees of drought stress treatment, 60‐day‐old plants were grown in the controlled environment as described above. Watering was stopped after plants were fully watered. Plants in pots were weighed at the start point and periodical time point of the drought stress treatment. Relative water contents of soil for the indicated treatments were 77% (well‐watered), 56% (mild drought), 25% (moderate drought), 11% (severe drought) and 77% (1 day after rewatering). Relative water content of the soil was determined gravimetrically by collecting 25 ml of soil around the area of the plant roots. The weight of the soil was determined as *W*_0_, and then the soil was oven‐dried at 65°C for 24 h, and weighed as *W*_t_. Relative soil water content was calculated using the following formula: RWC (%) = (*W*_0_ − *W*_t_)/*W*_0_ × 100%.

For ABA treatments, roots of 40‐day‐old tissue culture plantlets were soaked in a 100 μm ABA solution. A corresponding aqueous solution was used as the control.

### RNA extraction and quantitative real‐time PCR

Total RNA was extracted using TRIzol reagent (TaKaRa, Shiga‐ken, Japan). cDNAs were synthesized from 1 μg total RNA using the HiScript II One Step reverse transcription‐PCR kit (Vazyme, Nanjing, China). Quantitative real‐time PCR reactions were performed with an ABI StepOnePlus Real‐Time PCR system (Applied Biosystems, Foster City, CA, USA). The *CmUBIQUITIN* gene (GenBank accession EU862325) was used as the internal control. Expression was normalized to the reference *CmUBIQUITIN* gene using the comparative ΔΔ*C*_t_ method (Livak and Schmittgen, 2001). The primers used for gene expression analysis were listed in Table S3.

### Subcellular localization

The *CmBBX19* ORF sequence, without the stop codon, was cloned into the pSuper1300 (GFP‐C) vector driven by the *Super* promoter. Mesophyll protoplasts were prepared from 40‐day‐old chrysanthemum leaves according to Yoo *et al*. (2007), and 10 μg of *pSuper::CmBBX19‐GFP* plasmid, prepared using an OMEGA Plasmid Maxi Kit (OMEGA, Norcross, GA, USA), was introduced into approximately 2 × 10^4^ protoplasts with polyethylene glycol as described in Higuchi *et al*. (2013). Transformed mesophyll protoplasts were observed with an Olympus FV1000 confocal laser scanning microscope (Olympus, Tokyo, Japan) after being cultured for 16–20 h at 22°C. For confocal microscopy, GFP images were obtained with an excitation at 488 nm and emission at 525 nm. The primers used for vector construction were listed in Table S3.

### Chrysanthemum transformation

For construction of the overexpression vector, the *CmBBX19* ORF was cloned into the *Xba*I and *Sac*I sites of the pBI121 vector (Chen *et al*., 2003). For RNAi vector construction, a 319‐bp sense and antisense fragment of *CmBBX19* containing *Xho*I/*Cla*I and *Xba*I/*Kpn*I sites were cloned into the pHANNIBAL vector to obtain an intron containing “hairpin” RNA (ihpRNA) construct. The ihpRNA construct with 35S promoter and Nos terminator was then cloned into the binary pART27 vector (Wesley *et al*., 2001). These recombinant constructs were introduced separately into *Agrobacterium tumefaciens* strain EHA105, and then transformed into chrysanthemum (Hong *et al*., 2006).

### Drought stress treatment

The *CmBBX19*‐OX, *CmBBX19‐*RNAi and WT plants were transferred to 25‐cm pots containing 550 g of a 1:1 (v/v) mixture of peat and vermiculite, with three plants from each line per pot and grown in the controlled environment as described above. Plants were supplied with ample water and then water was retained for 30 days and recovery was performed for 5 days with regular watering. For the indicated treatments, relative water contents of soil were 77% (before treatment), 7% (drought 30 days) and 75% (rewatered 5 days). Survival rates were recorded after the drought treatments. The photographs were taken to record their phenotypes both before and after treatments.

### Measurements of transpiration rate, stomatal conductance, photosynthesis rate and water loss rate

Measurements of transpiration rate, stomatal conductance and photosynthesis rate of the top sixth expanded leaf were carried out using an LI‐6400XT Portable Photosynthesis System (LI‐CORE, Lincoln, NE, USA). Leaves were placed in a chamber at 22°C, 400 µmol mol^−1^ CO_2_ and 300 µmol m^−2^ sec^−1^ illumination. Data were recorded every 2 min once the infrared gas analyzer was stabilized.

The top fifth fully expanded leaves from 60‐day‐old *CmBBX19*‐OX, *CmBBX19*‐RNAi and WT plants were used for the analyses of relative water loss. Fresh weight (FW) of each leaf was measured immediately after detached from a plant. For the dehydration treatment, the desiccation weight (dW) of each leaf was measured at different time points. Finally, the leaf samples were dried at 65°C for 24 h to determine the dry weight (DW). Relative water loss rates were calculated using the following equation: relative water loss rate (%) = (FW − dW)/(FW − DW) × 100 (Fukao *et al*., 2011). The measurements were conducted with five biological replicates and the results are presented as the means ± SD.

### Quantification of endogenous ABA

Endogenous ABA levels in the pooled top four to five expanded leaves of transgenic chrysanthemum leaves were measured by extracting 50 mg FW tissue with methanol/water/formic acid (15:4:1, v/v/v). The extracts were dried by nitrogen gas stream, and redissolved in 100 μl 80% methanol (v/v), and filtered (polytetrafluoroethylene, 0.22 µm; Anpel, Shang Hai, China). ABA was detected using the AB Sciex QTRAP 6500 liquid chromatography‐tandem mass spectrometry (LC‐MS/MS) platform. AB 6500 QTRAP LC/MS/MS System was controlled by Analyst 1.6 software (AB Sciex, Waltham, MA, USA). The electrospray ionization source operation parameters, ion source, turbospray; source temperature, 500°C; ionspray voltage, 4500 V; curtain gas, 35.0 psi; the collision gas, medium. The measurements were performed using three biological replicates.

### RNA‐seq analysis

Total RNA was extracted from the top fifth expanded leaf from 60‐day‐old *CmBBX19*‐OX, *CmBBX19*‐RNAi and WT plants using TRIzol reagent (TaKaRa). RNA‐seq data were processed as described previously (Gao *et al*., 2016). Briefly, the low‐quality reads (*Q* value <20) and adapters were filtered using Trimmomatic (Bolger *et al*., 2014). High‐quality clean reads were *de novo* assembled into contigs using the Trinity program and iAssembler (Grabherr *et al*., 2011; Zheng *et al*., 2011). The assembled contigs were used in a search against the GenBank non‐redundant, UniProt and *A. thaliana* protein databases using BLAST with a cutoff E value of 1e^−5^.

### Dual‐luciferase reporter assay in *N. benthamiana*

For analysis of CmBBX19 transcriptional activation, the *CmBBX19* ORF sequence, without the stop codon, was cloned into the pBD‐VP16 vector (Han *et al*., 2016). The fusion constructs were introduced into *A. tumefaciens* strain GV3101, which was then shacked overnight. The *A. tumefaciens* were collected and adjusted to OD_600_ = 1.0 by infiltration buffer. *Agrobacterium tumefaciens* culture harboring *CmBBX19* was mixed with 1/5 volume of *A. tumefaciens* culture containing the reporter vector (Han *et al*., 2016), and infiltrated into *N. benthamiana* leaves using a needleless syringe.

To determine the *in vivo* interaction between CmBBX19/CmABF3 and the *CmRAB18* promoter, the *CmRAB18* promoter was inserted into the pGreenII 0800‐LUC vector, while the *CmBBX19* or *CmABF3* ORF sequences were cloned into the pGreenII 0029 62‐SK vector (Hellens *et al*., 2005). *Agrobacterium tumefaciens* strain harboring *CmBBX19*, *CmABF3* and *CmRAB18* promoter‐driven LUC constructs were mixed as 1:1:10 (v/v/v), and infiltrated into *N. benthamiana* leaves.

The dual‐luciferase reporter assay was conducted using the dual‐luciferase reporter assay systems (Promega, Madison, WI, USA) and d‐luciferin (Promega) as previously described (Gao *et al*., 2019). The LUC images were taken using an ikon‐L936 imaging system (Andor Tech, Belfast, UK). LUC and REN activities were determined using a GloMax 20/20 luminometer (Promega). The primers used for vector construction are listed in Table S3.

### Yeast two‐hybrid assays

The *CmBBX19* and *AtBBX19* ORF sequences were amplified and separately cloned into the *Eco*RI/*Sal*I sites of the pGBKT7 vector (Louvet *et al*., 1997). The ORF or ORF fragments of *CmABF* genes, *AtABF* genes and *AtABI5* were amplified and cloned into the *Eco*RI/*Xho*I sites of the pGADT7 vector (Chien *et al*., 1991). The pGADT7 and pGBKT7 recombinant plasmids were transformed into yeast strain Y2HGold together and the Y2H assay was conducted using the Matchmaker^TM^ GAL4 two‐hybrid system (Clontech, Shiga‐ken, Japan). Transformants were grown on SD/‐Trp‐Leu plates, and then transferred to SD/‐Trp‐Leu‐His‐Ade plates for spot assays. The PCR primers used for vector construction were listed in Table S3.

### BiFC

Constructs expressing CmBBX19‐YFP^N^, CmABF3‐YFP^C^ or control vectors were introduced into *A. tumefaciens* strain GV3101, which was then shaken overnight. The *A. tumefaciens* were collected and adjusted to OD_600_ = 1.0 by infiltration buffer. Combinations were co‐infiltrated into 4‐week‐old *N. benthamiana* leaves using a needleless syringe. The YFP fluorescence was imaged 60 h after infiltration using an Olympus FV1000 confocal laser scanning microscope. The excitation wavelength for YFP was 488 nm and emission wavelength was 525 nm. The primers used for vector construction were listed in Table S3.

### Yeast one‐hybrid assays

To construct bait vector, *CmRAB18* promoter fragments were amplified from chrysanthemum genomic DNA and then inserted into the *Kpn*I/*Sal*I sites of the pAbAi vector (Clontech). For prey construction, the *CmABF3* ORF was amplified and then inserted into the *Eco*RI/*Xho*I sites of the pGADT7 vector (Clontech). Y1H assays were performed using the Matchmaker^TM^ Gold Yeast One‐Hybrid Library Screening System (Clontech). Transformants were selected and grown on SD/‐Ura plates, and then transferred to SD/‐Ura‐Leu, and SD/‐Ura‐Leu+A, Aureobasidin A plates for spot assays. The PCR primers used for vector construction were listed in Table S3.

### EMSA

EMSA was conducted using biotin‐labeled probes and a Light Shift Chemiluminescent EMSA kit (Thermo Scientific, Waltham, MA, USA) as previously described (Dai *et al*., 2012). The *CmRAB18* promoter fragment (5′‐gAAATTAgACACgTACTTTTCAgTgATAACATAAACATACTTACgTgTTC‐3′) with a biotin‐labeled probe, and the same unlabeled fragment was used as a competitor. *Escherichia coli* Rosetta cells harboring GST‐CmBBX19 or GST‐CmABF3 recombinant plasmids were incubated at 16°C for 10 h to induce recombinant proteins by adding isopropylthio‐β‐galactoside to a final concentration of 0.4 mm. The recombinant proteins were purified using glutathione Sepharose 4B beads (GE Healthcare, Huston, TX, USA). Purified GST‐CmBBX19 and GST‐CmABF3 proteins were incubated with 2 nm biotin‐labeled probes in a 20 μl reaction mixture. The primers used for vector construction were listed in Table S3.

### Virus‐induced gene silencing

For *CmABF3* silencing in chrysanthemum, a previously reported virus‐based microRNA expression system was used (Tang *et al*., 2010). The candidate 21‐nt mature amiRNA‐CmABF3 sequence and primers for amplifying amiRNA‐CmABF3 were designed using the Web MicroRNA Designer (http://wmd3.weigelworld.org/cgi‐bin/webapp.cgi). amiR‐CmABF3 was cloned from the pRS300 plasmid into the CaLCuV vector, and then introduced into *A. tumefaciens* strain GV3101. *Agrobacterium tumefaciens* cells harboring recombinant plasmids were shaken overnight. The *A. tumefaciens* were collected and adjusted to OD_600_ = 1.5 by infiltration buffer. pCVB and CaLCuV (control) or CaLCuV‐CmABF3 were mixed in a 1:1 ratio (v/v), and were placed at 28°C in the dark for 3–4 h before infiltration. Forty‐day‐old tissue cultured chrysanthemum RNAi and WT plants were vacuumed in infiltration buffer for 3 min under 0.7 MPa, and then washed by deionized water. The plants were placed at 8°C in the dark for 3 days before transplanting in pots containing 50 g of a mixture of 1:1 (v/v) peat and vermiculite and growing under normal conditions (23 ± 1°C, 40% relative humidity, and 16 h light/8 h dark). Before drought stress treatment, the plants were validated by determination of the expression of *CmABF3*. Two independent experiments were conducted and at least 12 positive plantlets of WT or RNAi lines were used in each experiment.

For drought stress treatment, plants were fully watered and then water was withheld for 20 days. After treatment, the plants were photographed. Transpiration rate, stomatal conductance and photosynthesis rates were monitored as above, before treatment and after 20 days of drought.

## ACCESSION NUMBERS

Sequence data from this article can be found in the GenBank/EMBL databases under the following accession numbers: CmBBX19, *C. morifolium*, KP963930; CmBBX22a, *C. morifolium*, KP963929; CmBBX22b, *C. morifolium*, KP963932.1; CmBBX22c, *C. morifolium*, KP963928.1; CmBBX24, *C. morifolium*, KF385866; CmABF3, *C. morifolium*, MN885646; the raw sequence reads of RNA‐seq, *C. morifolium*, PRJNA598566.

## AUTHOR CONTRIBUTIONS

BH conceived, designed, and supported the experiments. YX and XZ performed most of the experiments; AP and JZ performed plant cultivation and vector construction; XM and MZ performed protein interaction assays; LC performed chrysanthemum transformation; JG and CM provided conceptual advice; YX and BH analyzed the data and wrote the manuscript.

## CONFLICT OF INTEREST

The authors have no conflict of interest to declare.

## Supporting information


**Figure S1**. Deduced CmBBX19 amino acid sequence analysis.

**Figure S2**. Expression levels of members of the BBX group IV in wild type (WT) and *CmBBX19‐*RNAi plants.

**Figure S3**. mRNA and protein levels of CmBBX19 in the overexpression lines.

**Figure S4**. ABA content in leaves of *CmBBX19* transgenic lines and wild type (WT).

**Figure S5**. Expression of genes related to abscisic acid (ABA) biosynthesis, signaling pathway, and leaf senescence in transgenic *CmBBX19‐*OX or *CmBBX19*‐RNAi chrysanthemum plants.

**Figure S6**. Distribution of ABRE and G‐box motifs in promoters of LEA protein genes, upregulated in *CmBBX19*‐RNAi plants.

**Figure S7**. Deduced amino acid sequence analysis of CmABF.

**Figure S8**. Analysis of BBX19‐ABF interaction in *Arabidopsis thaliana*.

**Figure S9**. Expression of abiotic stress‐responsive genes in the ABA‐dependent pathway in CaLCuV‐amiR‐ABF3‐infected *CmBBX19*‐RNAi plants.

**Table S1**. Expression profiles of BBX family subgroup IV genes in the chrysanthemum transcriptome database in response to dehydration.

**Table S2**. Differentially expressed genes related to abiotic stress tolerance in *CmBBX19* transgenic plants.

**Table S3**. Primers used for vector construction and quantitative real‐time PCR analysis.

**Methods S1**. Sequence analysis.

## References

[tpj14863-bib-0001] Bai, M.J. , Sun, J.J. , Liu, J.Y. , Ren, H.R. , Wang, K. , Wang, Y.L. , Wang, C.Q. and Dehesh, K. (2019) The B‐box protein BBX19 suppresses seed germination via induction of ABI5. Plant J. 99, 1192–1202. 31112314 10.1111/tpj.14415 PMC6744306

[tpj14863-bib-0002] Bolger, A.M. , Lohse, M. and Usadel, B. (2014) Trimmomatic, a flexible trimmer for Illumina sequence data. Bioinformatics, 30, 2114–2120. 24695404 10.1093/bioinformatics/btu170 PMC4103590

[tpj14863-bib-0003] Causier, B. , Ashworth, M. , Guo, W. and Davies, B. (2012) The TOPLESS interactome, a framework for gene repression in Arabidopsis. Plant Physiol. 158, 423–438. 22065421 10.1104/pp.111.186999 PMC3252085

[tpj14863-bib-0004] Chen, P.Y. , Wang, C.K. , Soong, S.C. and To, K.Y. (2003) Complete sequence of the binary vector pBI121 and its application in cloning T‐DNA insertion from transgenic plants. Mol. Breed. 11, 287–293.

[tpj14863-bib-0005] Chien, C.t. , Bartel, P.l. , Sternglanz, R. and Fields, S. (1991) The two‐hybrid system, a method to identify and clone genes for proteins that interact with a protein of interest. Proc. Natl. Acad. Sci. USA, 88, 9578–9582. 1946372 10.1073/pnas.88.21.9578 PMC52761

[tpj14863-bib-0006] Cutler, S.R. , Rodriguez, P.L. , Finkelstein, R.R. and Abrams, S.R. (2010) Abscisic acid, emergence of a core signaling network. Annu. Rev. Plant Biol. 61, 651–679. 20192755 10.1146/annurev-arplant-042809-112122

[tpj14863-bib-0007] Dai, F.W. , Zhang, C.Q. , Jiang, X.Q. , Kang, M. , Yin, X. , Lu, P.T. , Zhang, X. , Zheng, Y. and Gao, J.P. (2012) RhNAC2 and RhEXPA4 are involved in the regulation of dehydration tolerance during the expansion of rose petals. Plant Physiol. 160, 2064–2082. 23093360 10.1104/pp.112.207720 PMC3510132

[tpj14863-bib-0008] Dong, C.J. and Liu, J.Y. (2010) The Arabidopsis EAR‐motif‐containing protein RAP2.1 functions as an active transcriptional repressor to keep stress responses under tight control. BMC Plant Biol. 10, 47. 20230648 10.1186/1471-2229-10-47 PMC2848764

[tpj14863-bib-0009] Fujita, Y. , Fujita, M. , Satoh, R. , Maruyama, K. , Parvez, M.M. , Seki, M. , Hiratsu, K. , Ohme‐Takagi, M. , Shinozaki, K. and Yamaguchi‐Shinozaki, K. (2005) AREB1 is a transcription activator of novel ABRE‐dependent ABA signaling that enhances drought stress tolerance in Arabidopsis. Plant Cell, 17, 3470–3488. 16284313 10.1105/tpc.105.035659 PMC1315382

[tpj14863-bib-0010] Fujita, Y. , Fujita, M. , Shinozaki, K. and Yamaguchi‐Shinozaki, K. (2011) ABA‐mediated transcriptional regulation in response to osmotic stress in plants. J. Plant Res. 124, 509–525. 21416314 10.1007/s10265-011-0412-3

[tpj14863-bib-0053] Fukao, T. , Yeung, E. and Bailey‐Serres, J. (2011) The submergence tolerance regulator SUB1A mediates crosstalk between submergence and drought tolerance in rice. Plant Cell, 23(1), 412–427. 21239643 10.1105/tpc.110.080325 PMC3051255

[tpj14863-bib-0011] Furihata, T. , Maruyama, K. , Fujita, Y. , Umezawa, T. , Yoshida, R. , Shinozaki, K. and Yamaguchi‐Shinozaki, K. (2006) Abscisic acid‐dependent multisite phosphorylation regulates the activity of a transcription activator AREB1. Proc. Natl Acad. Sci. USA, 103, 1988–1993. 16446457 10.1073/pnas.0505667103 PMC1413621

[tpj14863-bib-0012] Gangappa, S.N. and Botto, J.F. (2014) The BBX family of plant transcription factors. Trends Plant Sci. 19, 460–470. 24582145 10.1016/j.tplants.2014.01.010

[tpj14863-bib-0013] Gao, Y.R. , Liu, C. , Li, X.D. , Xu, H.Q. , Liang, Y. , Ma, N. , Fei, Z.J. , Gao, J.P. , Jiang, C.Z. and Ma, C. (2016) Transcriptome profiling of petal abscission zone and functional analysis of an Aux/IAA family gene RhIAA16 involved in petal shedding in rose. Front. Plant Sci. 7, 1375. 27695465 10.3389/fpls.2016.01375 PMC5023668

[tpj14863-bib-0014] Gao, Y.R. , Liu, Y. , Liang, Y. , Lu, J.Y. , Jiang, C.Y. , Fei, Z.J. , Jiang, C.Z. , Ma, C. and Gao, J.P. (2019) Rosa hybrida RhERF1 and RhERF4 mediate ethylene and auxin‐regulated petal abscission by influencing pectin degradation. Plant J. 10, 1111/tpj.14412. 10.1111/tpj.14412 31111587

[tpj14863-bib-0015] Grabherr, M.G. , Haas, B.J. , Yassour, M. ***et al*** (2011) Full‐length transcriptome assembly from RNA‐Seq data without a reference genome. Nat. Biotechnol. 29, 644–652. 21572440 10.1038/nbt.1883 PMC3571712

[tpj14863-bib-0016] Han, Y.C. , Kuang, J.F. , Chen, J.Y. , Liu, X.C. , Xiao, Y.Y. , Fu, C.C. , Wang, J.N. , Wu, K.Q. and Lu, W.J. (2016) Banana transcription factor MaERF11 recruits histone deacetylase MaHDA1 and represses the expression of MaACO1 and expansins during fruit ripening. Plant Physiol. 171, 1070–1084. 27208241 10.1104/pp.16.00301 PMC4902611

[tpj14863-bib-0017] Hellens, R.P. , Allan, A.C. , Friel, E.N. , Bolitho, K. , Grafton, K. , Templeton, M.D. , Karunairetnam, S. , Gleave, A.P. and Laing, W.A. (2005) Transient expression vectors for functional genomics., quantification of promoter activity and RNA silencing in plants. Plant Methods, 1, 13. 16359558 10.1186/1746-4811-1-13 PMC1334188

[tpj14863-bib-0018] Higuchi, Y. , Narumi, T. , Oda, A. , Nakano, Y. , Sumitomo, K. , Fukai, S. and Hisamatsu, T. (2013) The gated induction system of a systemic floral inhibitor., antiflorigen., determines obligate short‐day flowering in chrysanthemums. Proc. Natl Acad. Sci. USA, 110, 17137–17142. 24082137 10.1073/pnas.1307617110 PMC3801008

[tpj14863-bib-0019] Hong, B. , Tong, Z. , Ma, N. , Kasuga, M. , Yamaguchi‐Shinozaki, K. and Gao, J.P. (2006) Expression of the Arabidopsis DREB1A gene in transgenic chrysanthemum enhances tolerance to low temperature. J. Hortic. Sci. Biotechnol. 81, 1002–1008.

[tpj14863-bib-0020] Jakoby, M. , Weisshaar, B. , Droge‐Laser, W. , Vicente‐Carbajosa, J. , Tiedemann, J. , Kroj, T. and Parcy, F. (2002) bZIP transcription factors in Arabidopsis. Trends Plant Sci. 7, 106–111. 11906833 10.1016/s1360-1385(01)02223-3

[tpj14863-bib-0021] Kagale, S. and Rozwadowski, K. (2011) EAR motif‐mediated transcriptional repression in plants, an underlying mechanism for epigenetic regulation of gene expression. Epigenetics, 6, 141–146. 20935498 10.4161/epi.6.2.13627 PMC3278782

[tpj14863-bib-0022] Kang, X. , Xu, G. , Lee, B. , Chen, C. , Zhang, H. , Kuang, R. and Ni, M. (2018) HRB2 and BBX21 interaction modulates Arabidopsis ABI5 locus and stomatal aperture. Plant, Cell Environ. 41, 1912–1925. 29748960 10.1111/pce.13336

[tpj14863-bib-0023] Kazan, K. (2006) Negative regulation of defence and stress genes by EAR‐motif‐containing repressors. Trends Plant Sci. 11, 109–112. 16473545 10.1016/j.tplants.2006.01.004

[tpj14863-bib-0024] Khanna, R. , Kronmiller, B. , Maszle, D.R. , Coupland, G. , Holm, M. , Mizuno, T. and Wu, S.H. (2009) The Arabidopsis B‐box zinc finger family. Plant Cell, 21, 3416–3420. 19920209 10.1105/tpc.109.069088 PMC2798317

[tpj14863-bib-0027] Klug, A. and Schwabe, J.W.R. (1995) Zinc fingers. FASEB J. 9, 597–604. 7768350

[tpj14863-bib-0028] Livak, K.J. and Schmittgen, T.D. (2001) Analysis of relative gene expression data using real ‐time quantitative PCR and the 2^−ΔΔCT^ method. Methods, 25, 402–408. 11846609 10.1006/meth.2001.1262

[tpj14863-bib-0029] Liu, Y.N. , Chen, H. , Ping, Q. , Zhang, Z.X. , Guan, Z.Y. , Fang, W.M. , Chen, S.M. , Chen, F.D. , Jiang, J.F. and Zhang, F. (2019) The heterologous expression of CmBBX22 delays leaf senescence and improves drought tolerance in Arabidopsis. Plant Cell Rep. 38, 15–24. 30238422 10.1007/s00299-018-2345-y

[tpj14863-bib-0030] Louvet, O. , Doignon, F. and Crouzet, M. (1997) Stable DNA‐binding yeast vector allowing high‐bait expression for use in the two‐hybrid system. Biotechniques, 23, 816–820. 9383543 10.2144/97235bm11

[tpj14863-bib-0031] Nagaoka, S. and Takano, T. (2003) Salt tolerance‐related protein STO binds to a Myb transcription factor homologue and confers salt tolerance in Arabidopsis. J. Exp. Bot. 54, 2231–2237. 12909688 10.1093/jxb/erg241

[tpj14863-bib-0032] Raghavendra, A.S. , Gonugunta, V.K. , Christmann, A. and Grill, E. (2010) ABA perception and signalling. Trends Plant Sci. 15, 395–401. 20493758 10.1016/j.tplants.2010.04.006

[tpj14863-bib-0033] Tang, Y. , Wang, F. , Zhao, J. , Xie, K. , Hong, Y.G. and Liu, Y.L. (2010) Virus‐based microRNA expression for gene functional analysis in plants. Plant Physiol. 153, 632–641. 20388670 10.1104/pp.110.155796 PMC2879806

[tpj14863-bib-0035] Uno, Y. , Furihata, T. , Abe, H. , Yoshida, R. , Shinozaki, K. and Yamaguchi‐Shinozaki, K. (2000) Arabidopsis basic leucine zipper transcription factors involved in an abscisic acid‐dependent signal transduction pathway under drought and high‐salinity conditions. Proc. Natl Acad. Sci. USA, 97, 11632–11637. 11005831 10.1073/pnas.190309197 PMC17252

[tpj14863-bib-0036] Vaishak, K.P. , Yadukrishnan, P. , Bakshi, S. , Kushwaha, A.K. , Ramachandran, H. , Job, N. , Babu, D. and Datta, S. (2019) The B‐box bridge between light and hormones in plants. J. Photochem. Photobiol. B, 191, 164–174. 30640143 10.1016/j.jphotobiol.2018.12.021

[tpj14863-bib-0037] Wang, C.Q. , Guthrie, C. , Sarmast, M.K. and Dehesh, K. (2014) BBX19 interacts with CONSTANS to repress FLOWERING LOCUS T transcription, defining a flowering time checkpoint in Arabidopsis. Plant Cell, 26, 3589–3602. 25228341 10.1105/tpc.114.130252 PMC4213167

[tpj14863-bib-0039] Wang, Q.M. , Tu, X.J. , Zhang, J.H. , Chen, X.B. and Rao, L.J. (2013) Heat stress‐induced BBX18 negatively regulates the thermotolerance in Arabidopsis. Mol. Biol. Rep. 40, 2679–2688. 23238922 10.1007/s11033-012-2354-9

[tpj14863-bib-0040] Wei, Q. , Ma, C. , Xu, Y.J. , Wang, T.L. , Chen, Y.Y. , Lu, J. , Zhang, L.L. , Jiang, C.Z. , Hong, B. and Gao, J.P. (2017) Control of chrysanthemum flowering through integration with an aging pathway. Nat. Commun. 8, 829. 29018260 10.1038/s41467-017-00812-0 PMC5635119

[tpj14863-bib-0041] Wesley, S.V. , Helliwell, C.A. , Smith, N.A. ***et al*** (2001) Construct design for efficient, effective and high‐throughput gene silencing in plants. Plant J. 27, 581–590. 11576441 10.1046/j.1365-313x.2001.01105.x

[tpj14863-bib-0042] Xu, D. , Li, J. , Gangappa, S.N. , Hettiarachchi, C. , Lin, F. , Andersson, M.X. , Jiang, Y. , Deng, X.W. and Holm, M. (2014) Convergence of Light and ABA signaling on the ABI5 promoter. PLoS Genet. 10, e1004197. 24586210 10.1371/journal.pgen.1004197 PMC3937224

[tpj14863-bib-0043] Xu, Y.J. , Gao, S. , Yang, Y.J. , Huang, M.Y. , Cheng, L.N. , Wei, Q. , Fei, Z.J. , Gao, J.P. and Hong, B. (2013) Transcriptome sequencing and whole genome expression profiling of chrysanthemum under dehydration stress. BMC Genom. 14, 662. 10.1186/1471-2164-14-662 PMC3849779 24074255

[tpj14863-bib-0044] Yamaguchi‐Shinozaki, K. and Shinozaki, K. (2006) Transcriptional regulatory networks in cellular responses and tolerance to dehydration and cold stresses. Annu. Rev. Plant Biol. 57, 781–803. 16669782 10.1146/annurev.arplant.57.032905.105444

[tpj14863-bib-0045] Yang, Y.J. , Ma, C. , Xu, Y.J. ***et al*** (2014) A zinc finger protein regulates flowering time and abiotic stress tolerance in chrysanthemum by modulating gibberellin biosynthesis. Plant Cell, 26, 2038–2054. 24858937 10.1105/tpc.114.124867 PMC4079367

[tpj14863-bib-0046] Yoo, S.D. , Cho, Y.H. and Sheen, J. (2007) Arabidopsis mesophyll protoplasts, a versatile cell system for transient gene expression analysis. Nat. Protoc. 2, 1565–1572. 17585298 10.1038/nprot.2007.199

[tpj14863-bib-0047] Yoshida, T. , Fujita, Y. , Maruyama, K. , Mogami, J. , Todaka, D. , Shinozaki, K. and Yamaguchi‐Shinozaki, K. (2015) Four Arabidopsis AREB/ABF transcription factors function predominantly in gene expression downstream of SnRK2 kinases in abscisic acid signalling in response to osmotic stress. Plant, Cell Environ. 38, 35–49. 24738645 10.1111/pce.12351 PMC4302978

[tpj14863-bib-0048] Yoshida, T. , Fujita, Y. , Sayama, H. , Kidokoro, S. , Maruyama, K. , Mizoi, J. , Shinozaki, K. and Yamaguchi‐Shinozaki, K. (2010) AREB1, AREB2, and ABF3 are master transcription factors that cooperatively regulate ABRE‐dependent ABA signaling involved in drought stress tolerance and require ABA for full activation. Plant J. 61, 672–685. 19947981 10.1111/j.1365-313X.2009.04092.x

[tpj14863-bib-0049] Yoshida, T. , Mogami, J. and Yamaguchi‐Shinozaki, K. (2014) ABA‐dependent and ABA‐independent signaling in response to osmotic stress in plants. Curr. Opin. Plant Biol. 21, 133–139. 25104049 10.1016/j.pbi.2014.07.009

[tpj14863-bib-0050] Yuan, X. , Huang, P. , Wang, R.Q. , Li, H.Y. , Lv, X.Q. , Duan, M. , Tang, H.J. , Zhang, H.S. and Huang, J. (2018) A zinc finger transcriptional repressor confers pleiotropic effects on rice growth and drought tolerance by down‐regulating stress‐responsive genes. Plant Cell Physiol. 59, 2129–2142. 30020522 10.1093/pcp/pcy133

[tpj14863-bib-0051] Zhao, X. , Dou, L. , Gong, Z. , Wang, X. and Mao, T. (2019) BES1 hinders ABSCISIC ACID INSENSITIVE5 and promotes seed germination in Arabidopsis. New Phytol. 221, 908–918. 30230549 10.1111/nph.15437

[tpj14863-bib-0052] Zheng, Y. , Zhao, L.J. , Gao, J.P. and Fei, Z.J. (2011) iAssembler, a package for de novo assembly of Roche‐454/Sanger transcriptome sequences. BMC Bioinformatics, 12, 453. 22111509 10.1186/1471-2105-12-453 PMC3233632

