# Supplementary figures and images for: A zinc finger protein BBX19 interacts with ABF3 to affect drought tolerance negatively in chrysanthemum
Source: Plant J. 2020 Jul 21;103(5):1783–95. doi: 10.1111/tpj.14863 (PMC7496117; doi:10.1111/tpj.14863)

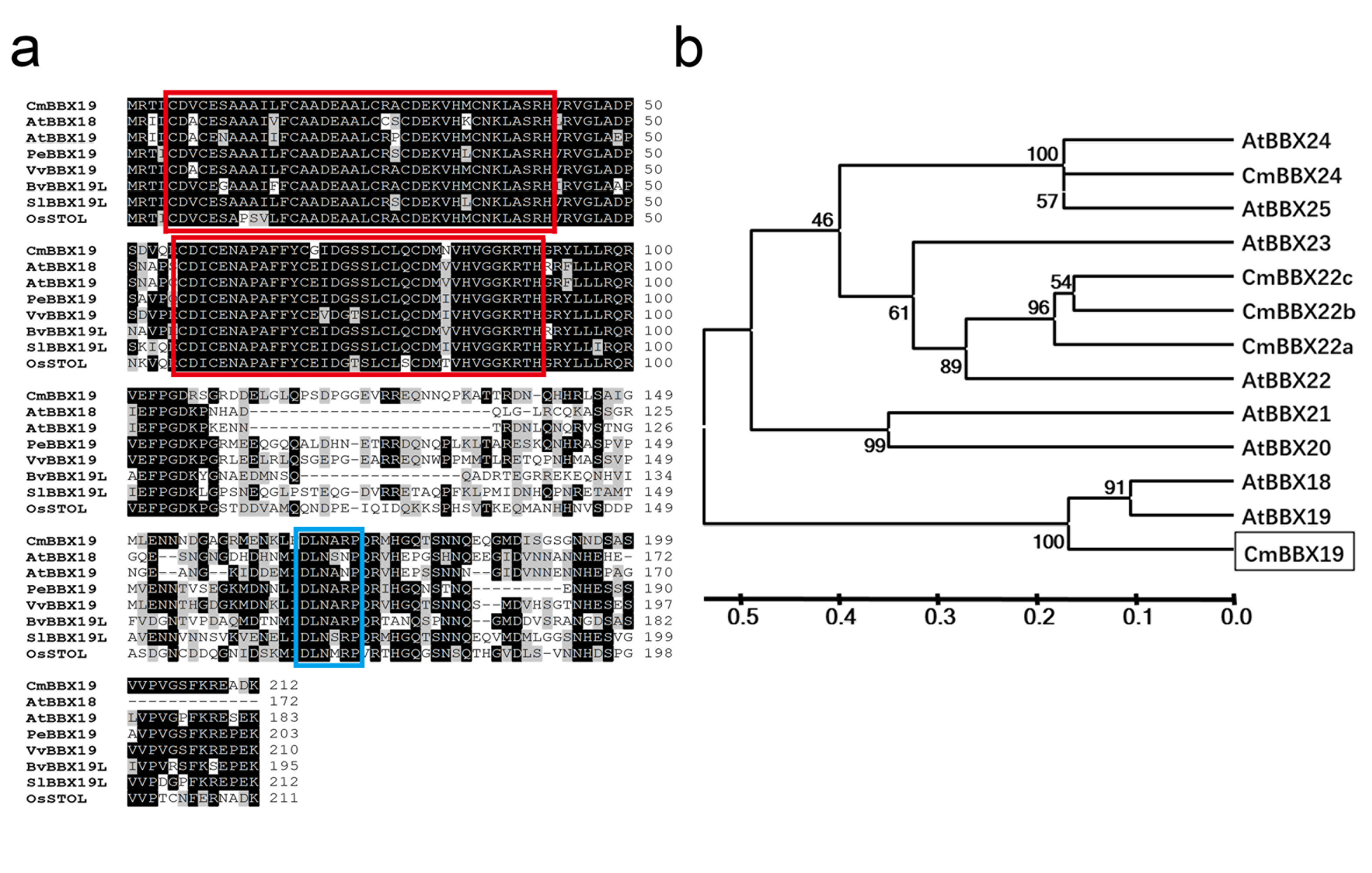

Supplement: Supplementary file 1 — Figure S1. Deduced CmBBX19 amino acid sequence analysis. [file TPJ-103-1783-s001.tif]

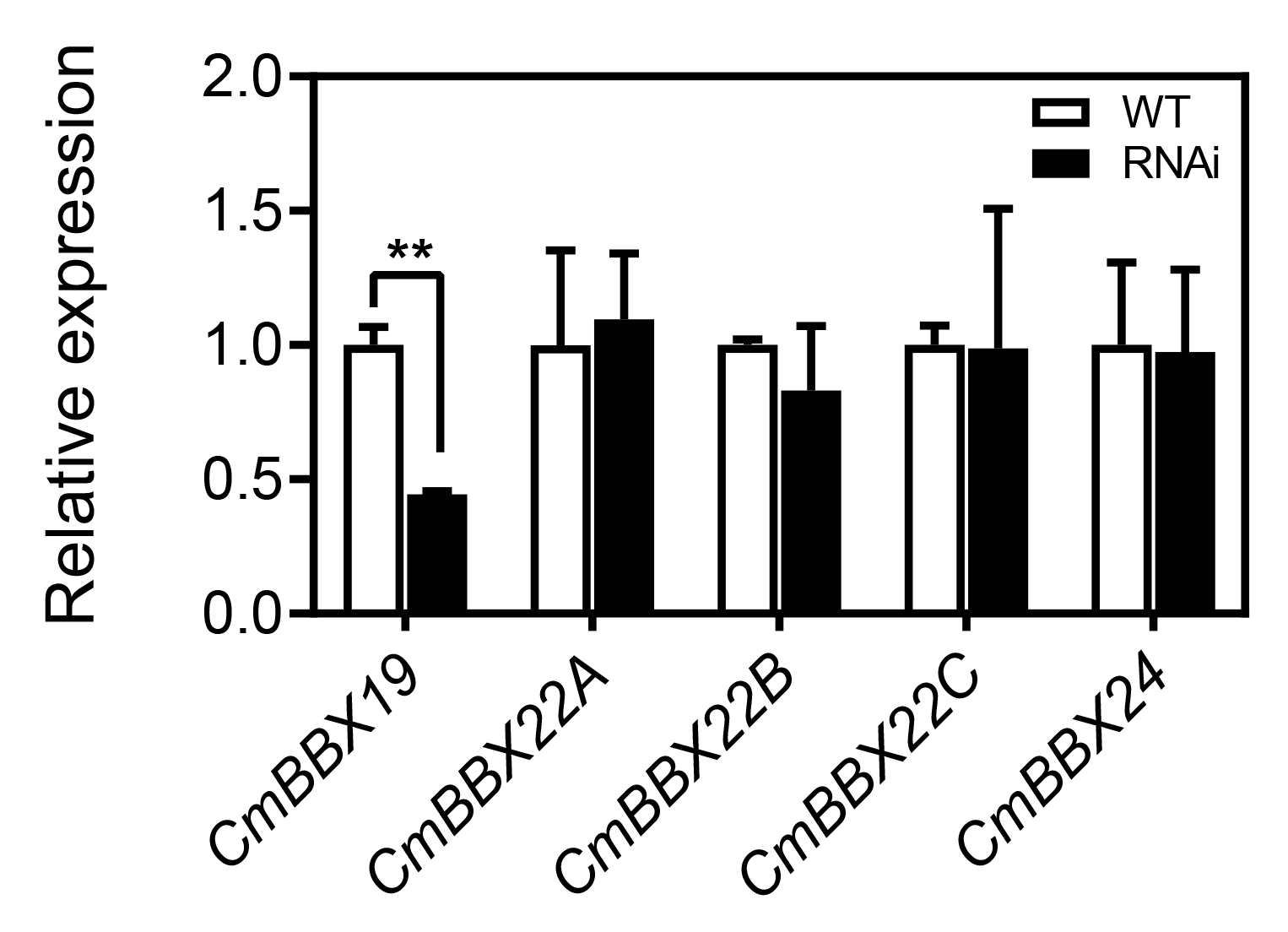

Supplement: Supplementary file 2 — Figure S2. Expression levels of members of the BBX group IV in wild type (WT) and CmBBX19‐RNAi plants. [file TPJ-103-1783-s002.tif]

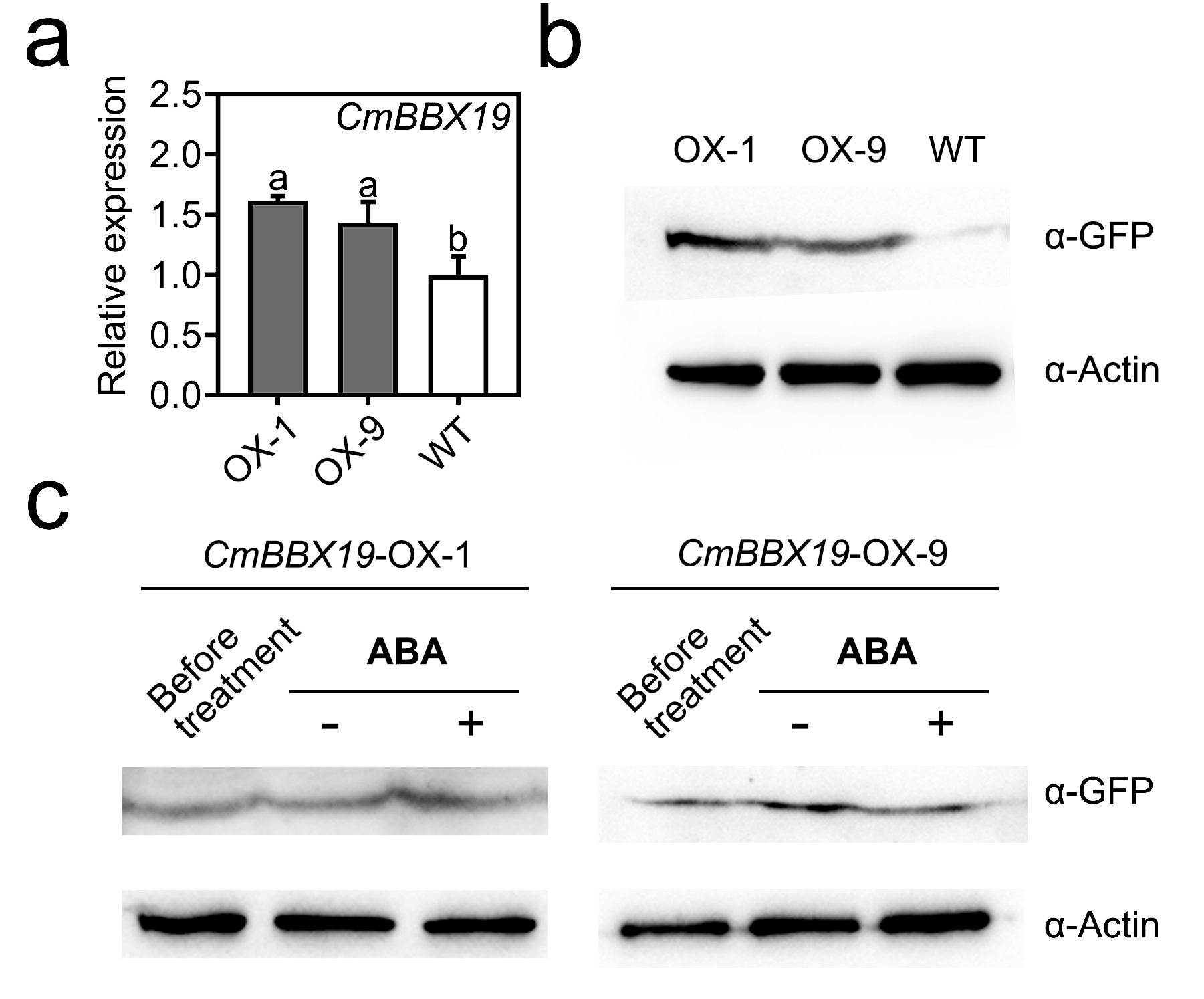

Supplement: Supplementary file 3 — Figure S3. mRNA and protein levels of CmBBX19 in the overexpression lines. [file TPJ-103-1783-s003.tif]

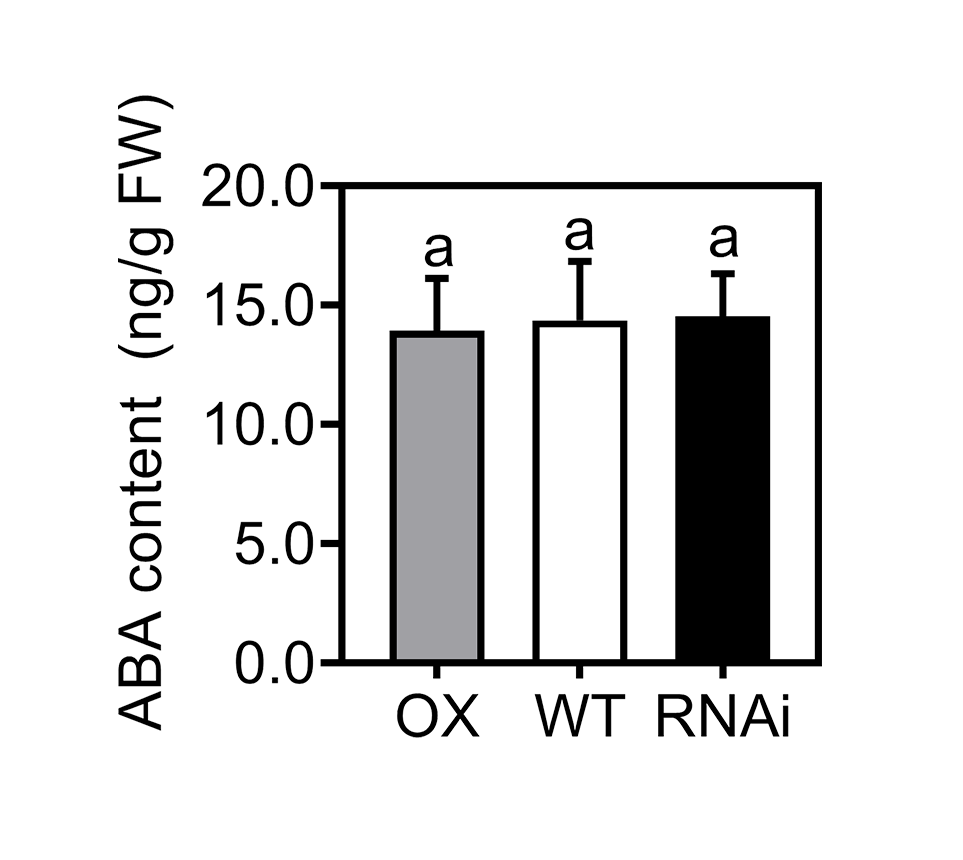

Supplement: Supplementary file 4 — Figure S4. ABA content in leaves of CmBBX19 transgenic lines and wild type (WT). [file TPJ-103-1783-s004.tif]

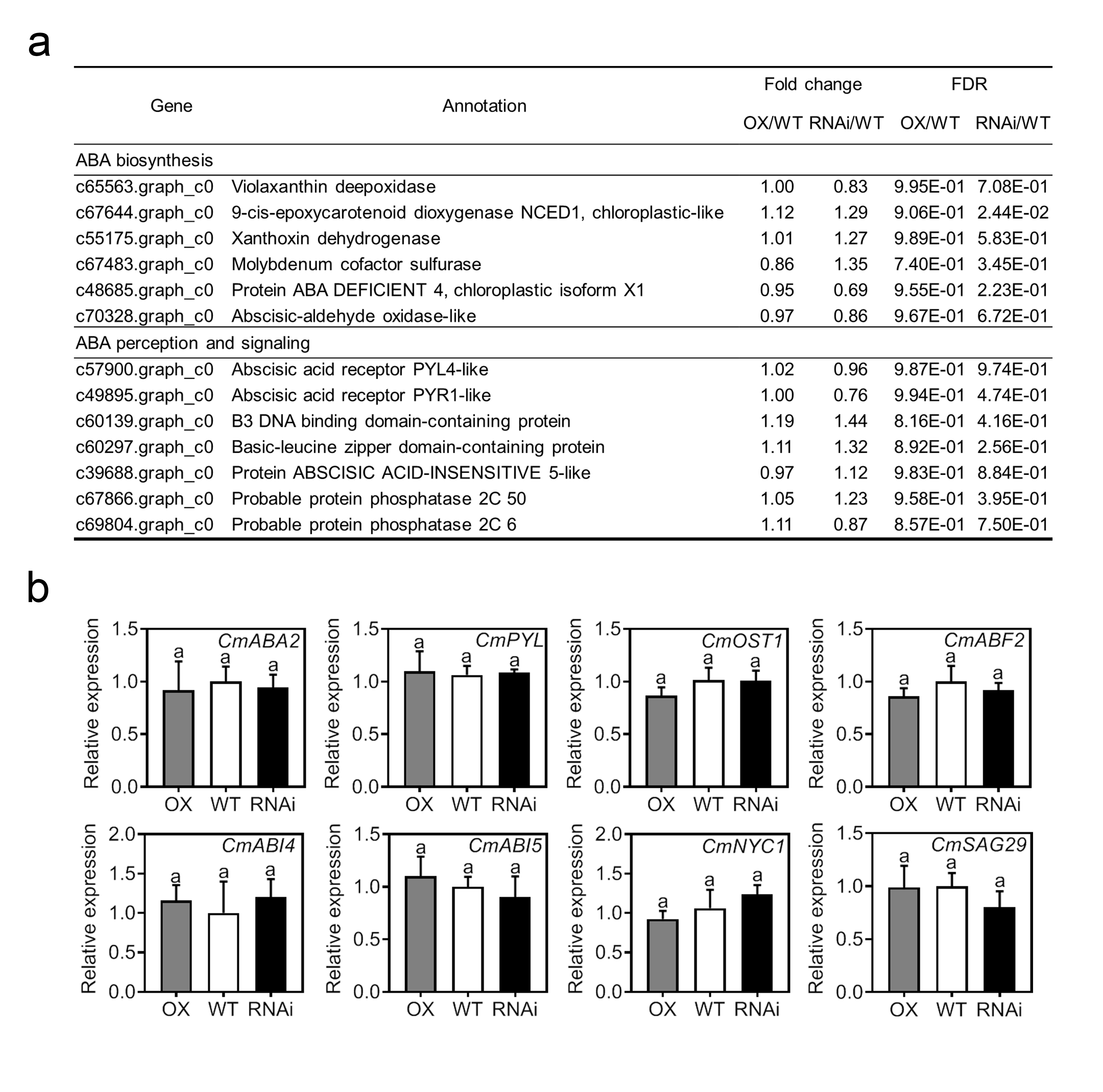

Supplement: Supplementary file 5 — Figure S5. Expression of genes related to abscisic acid (ABA) biosynthesis, signaling pathway, and leaf senescence in transgenic CmBBX19‐OX or CmBBX19‐RNAi chrysanthemum plants. [file TPJ-103-1783-s005.tif]

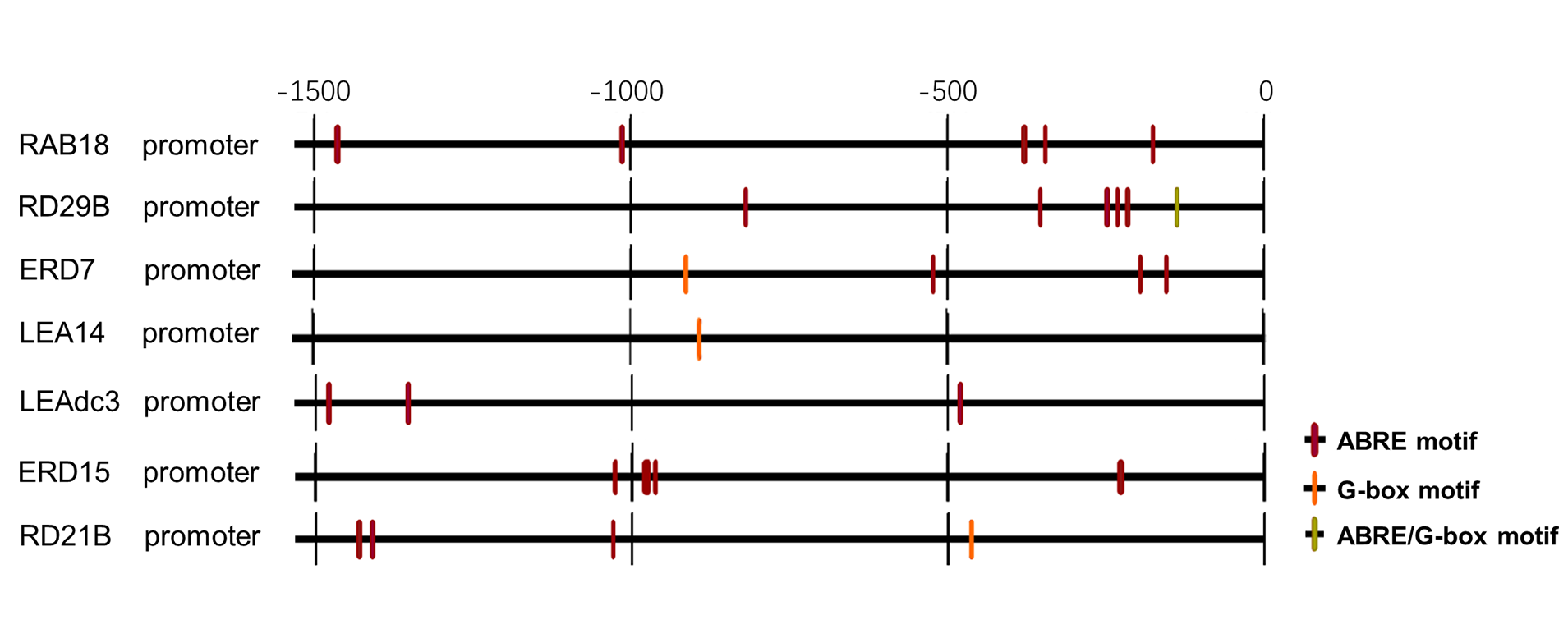

Supplement: Supplementary file 6 — Figure S6. Distribution of ABRE and G‐box motifs in promoters of LEA protein genes, upregulated in CmBBX19‐RNAi plants. [file TPJ-103-1783-s006.tif]

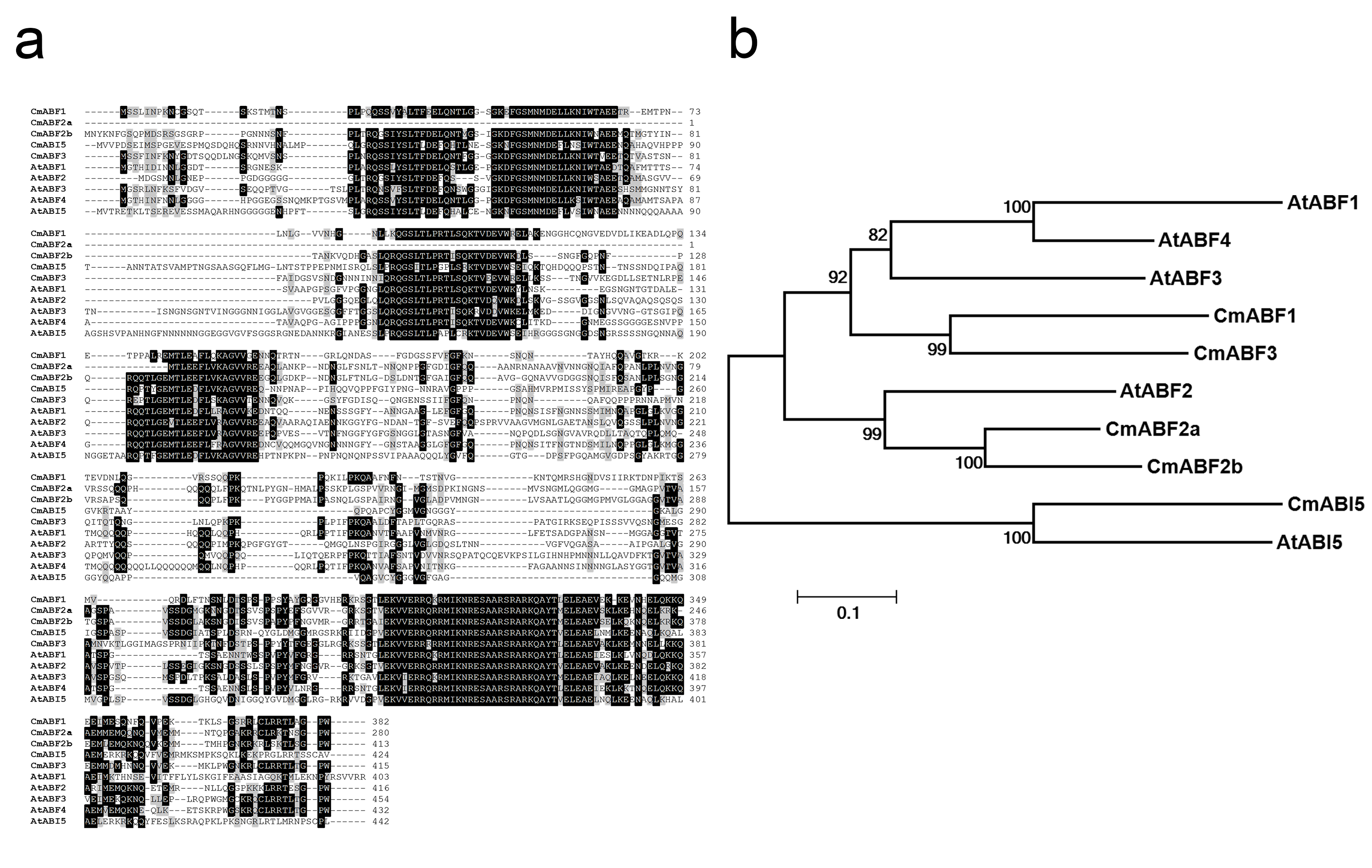

Supplement: Supplementary file 7 — Figure S7. Deduced amino acid sequence analysis of CmABF. [file TPJ-103-1783-s007.tif]

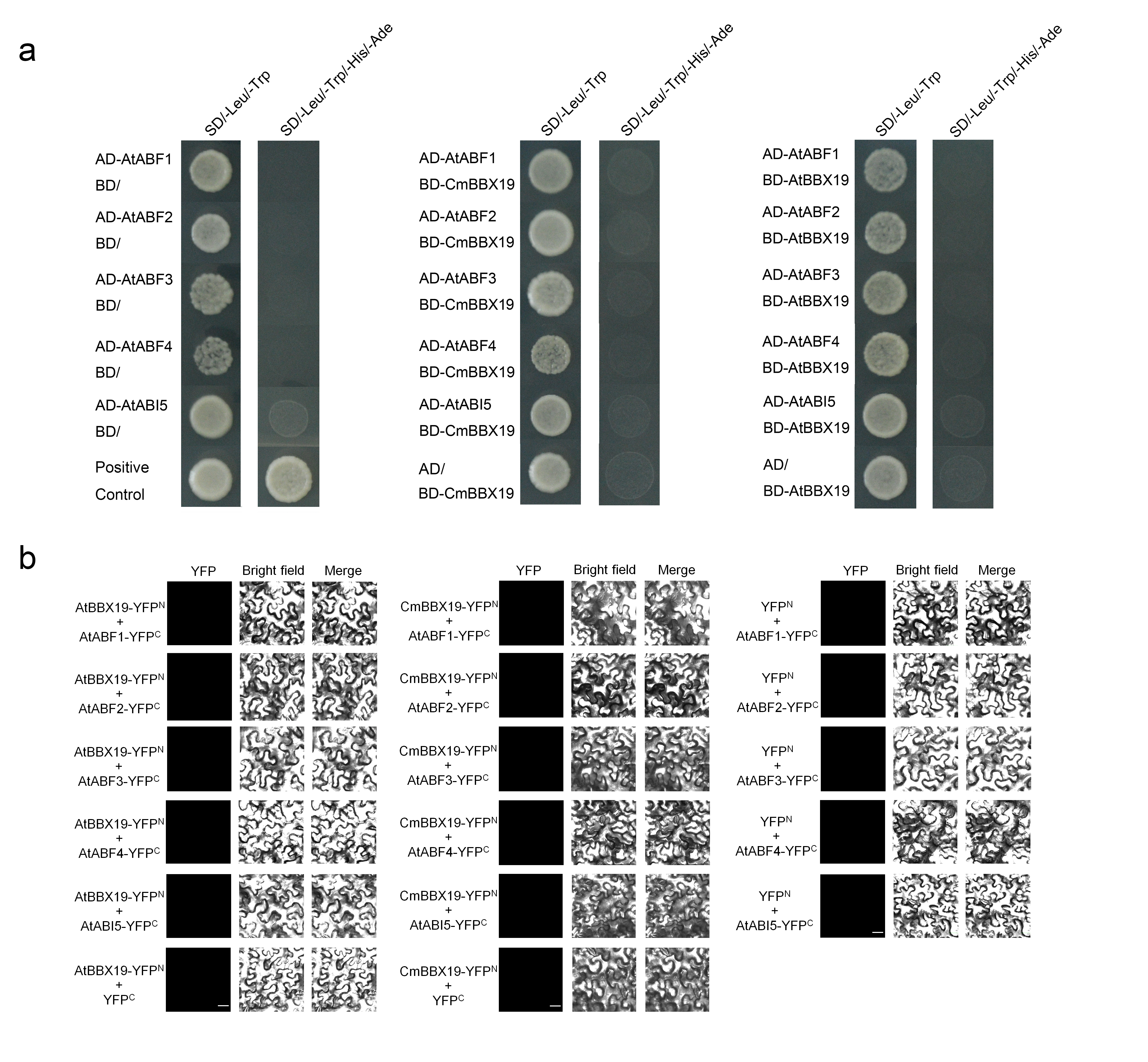

Supplement: Supplementary file 8 — Figure S8. Analysis of BBX19‐ABF interaction in Arabidopsis thaliana. [file TPJ-103-1783-s008.tif]

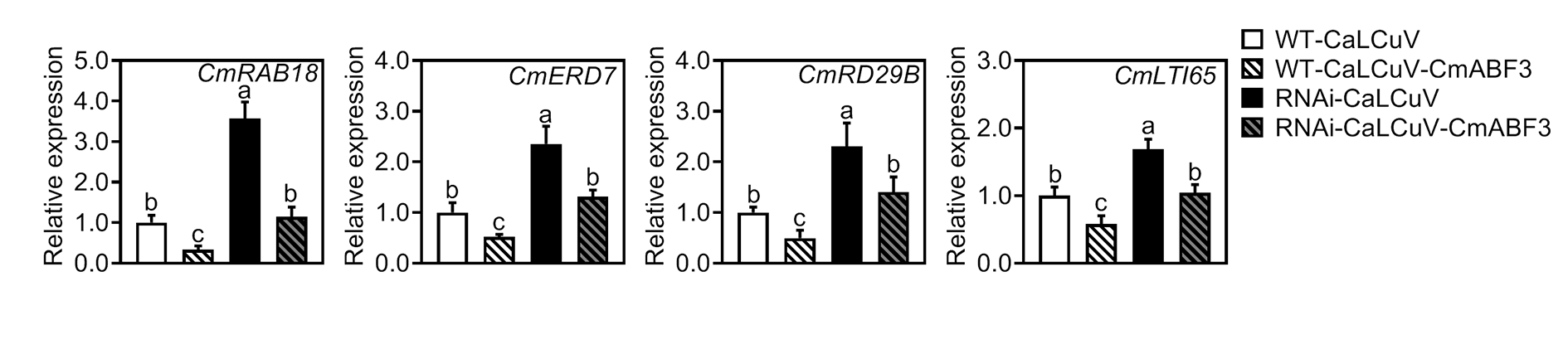

Supplement: Supplementary file 9 — Figure S9. Expression of abiotic stress‐responsive genes in the ABA‐dependent pathway in CaLCuV‐amiR‐ABF3‐infected CmBBX19‐RNAi plants. [file TPJ-103-1783-s009.tif]
