# Supplementary material for: A zinc finger protein BBX19 interacts with ABF3 to affect drought tolerance negatively in chrysanthemum
Source: Plant J. 2020 Jul 21;103(5):1783–95. doi: 10.1111/tpj.14863 (PMC7496117; doi:10.1111/tpj.14863)
Supplement: Supplementary file 10 — Table S1. Expression profiles of BBX family subgroup IV genes in the chrysanthemum transcriptome database in response to dehydration. [file TPJ-103-1783-s010.docx]

**Table S1.** Expression profiles of BBX family subgroup IV genes in the chrysanthemum transcriptome database in response to dehydration.

| **GeneID** | **BlastX ID** | **Con^a^** | **Deh^a^** | **Deh/Con**  **fold change** | **P-value** |
| --- | --- | --- | --- | --- | --- |
| UN09246 | XP_002316844 | 11.23 | 11.33 | 1.01 | 9.33E-06 |
| UN56679 | XP_002306904 | 9.02 | 16.72 | 1.85 | 4.81E-03 |
| **UN68402** | **XP_002267957** | **87.83** | **43.72** | **0.50** | **1.16E-03** |
| UN78385 | XP_002524543 | 6.42 | 24.58 | 3.83 | 4.06E-05 |

Con: control samples; Deh: dehydration treated samples. **a** Numbers in the columns are RPKM (reads per kilobase of exon model per million mapped reads) values.
