# Supplementary material for: A zinc finger protein BBX19 interacts with ABF3 to affect drought tolerance negatively in chrysanthemum
Source: Plant J. 2020 Jul 21;103(5):1783–95. doi: 10.1111/tpj.14863 (PMC7496117; doi:10.1111/tpj.14863)
Supplement: Supplementary file 11 — Table S2. Differentially expressed genes related to abiotic stress tolerance in CmBBX19 transgenic plants. [file TPJ-103-1783-s011.docx]

**Table S2.** Differentially expressed genes related to abiotic stress tolerance in *CmBBX19* transgenic plants.

| Gene | Annotation | Fold change | | | FDR | |
| --- | --- | --- | --- | --- | --- | --- |
|  |  | OX/WT | RNAi/WT | OX/WT | | RNAi/WT |
| **Signal proteins** | | | | | | |
| Transcription factor | | | | | | |
| c65640.graph_c0 | NAC domain-containing protein 101-like | 0.97 | 3.76 | 9.82E-01 | | 8.93E-04 |
| c58925.graph_c0 | ERF/AP-2 like transcription factor | 1.21 | 5.06 | 5.65E-01 | | 1.35E-03 |
| c62585.graph_c0 | Ethylene-responsive transcription factor 3-like, ERF3 | 1.10 | 5.28 | 9.07E-01 | | 3.48E-04 |
| c68561.graph_c0 | Ethylene-responsive transcription factor 5, ERF5 | 0.93 | 4.06 | 9.49E-01 | | 2.25E-05 |
| c54215.graph_c0 | Ethylene-responsive transcription factor 6, ERF6 | 0.82 | 2.95 | 8.01E-01 | | 9.04E-03 |
| c62496.graph_c0 | Zinc finger protein ZAT10-like | 1.04 | 6.06 | 9.80E-01 | | 1.63E-04 |
| c62716.graph_c0 | Zinc finger protein, ZAT10 | 1.13 | 5.62 | 7.95E-01 | | 1.78E-04 |
| Kinase | | | | | | |
| c67064.graph_c0 | Wall-associated receptor kinase 2-like, WAK2 | 0.81 | 4.38 | 7.09E-01 | | 3.05E-03 |
| c62503.graph_c0 | Cysteine-rich receptor-like protein kinase 10, CRK25 | 0.73 | 4.11 | 5.72E-01 | | 1.35E-03 |
| c61603.graph_c0 | Cysteine-rich receptor-like protein kinase 2, CRK2 | 0.57 | 3.03 | 3.22E-02 | | 9.46E-03 |
| c32781.graph_c0 | Cysteine-rich receptor-like protein kinase 2, CRK3 | 0.74 | 6.28 | 5.34E-01 | | 1.51E-05 |
| **Function protein** | | | | | | |
| LEA and dehydration response | | | | | | |
| c52943.graph_c0 | RESPONSIVE TO ABA 18, RAB18 | 0.67 | 2.72 | 3.20E-01 | | 1.42E-02 |
| c64947.graph_c0 | Low-temperature-induced 65 kDa protein-like, LTI65 | 1.02 | 2.43 | 9.81E-01 | | 6.41E-03 |
| c60013.graph_c0 | Protein EARLY-RESPONSIVE TO DEHYDRATION 7, ERD7 | 1.36 | 4.53 | 2.51E-01 | | 6.11E-05 |
| c64010.graph_c0 | Late embryogenesis abundant protein | 0.97 | 5.06 | 9.82E-01 | | 1.20E-03 |
| c60333.graph_c0 | Late embryogenesis abundant protein, LEA-14 | 1.03 | 3.01 | 9.80E-01 | | 5.17E-03 |
| c63880.graph_c0 | NDR1/HIN1-like protein 13, NHL13 | 1.09 | 4.89 | 9.38E-01 | | 1.24E-03 |
| c60127.graph_c0 | Protein EARLY RESPONSIVE TO DEHYDRATION 15, ERD15 | 0.94 | 6.45 | 9.58E-01 | | 2.19E-05 |
| c58132.graph_c0 | Probable cysteine protease, RD21B | 0.92 | 2.64 | 8.79E-01 | | 1.42E-07 |
| Oxidation-reduction | | | | | | |
| c62219.graph_c0 | Peroxidase N1-like | 0.86 | 4.40 | 7.93E-01 | | 2.65E-03 |
| c44553.graph_c0 | Peroxidase 12-like | 0.57 | 2.52 | 8.73E-02 | | 6.96E-04 |
| c52403.graph_c0 | Thioredoxin-like 3-1, chloroplastic | 0.69 | 2.02 | 5.33E-02 | | 2.43E-03 |
| c37240.graph_c0 | Thioredoxin | 1.30 | 3.48 | 8.48E-01 | | 4.14E-04 |
| c49215.graph_c1 | Thioredoxin-like protein CXXS1 | 0.54 | 4.68 | 5.63E-01 | | 1.04E-03 |
| c45050.graph_c0 | Glutaredoxin-C11-like | 1.31 | 4.24 | 7.64E-01 | | 2.29E-04 |
| c63036.graph_c0 | Protein DETOXIFICATION 49-like | 1.40 | 10.12 | 7.00E-01 | | 3.55 E-04 |
| Cell wall biogenesis | | | | | | |
| c65258.graph_c0 | Cellulose synthase-like protein G2 | 0.35 | 4.08 | 9.55E-06 | | 6.92E-12 |
| c44184.graph_c0 | Glycine-rich cell wall structural protein 1-like | 1.35 | 2.21 | 7.54E-01 | | 4.28E-03 |
| c49961.graph_c0 | Probable xyloglucan endotransglucosylase/hydrolase protein 23 | 1.44 | 14.58 | 9.31E-01 | | 6.00E-06 |

Note: OX, *CmBBX19*-overexpressed plant; RNAi, *CmBBX19*-RNAi plant; WT, wild type plant. Significant differences were corrected with P<0.05 and expression ratio ≥ 1.5.
