# Supplementary material for: A zinc finger protein BBX19 interacts with ABF3 to affect drought tolerance negatively in chrysanthemum
Source: Plant J. 2020 Jul 21;103(5):1783–95. doi: 10.1111/tpj.14863 (PMC7496117; doi:10.1111/tpj.14863)
Supplement: Supplementary file 12 — Table S3. Primers used for vector construction and quantitative real‐time PCR analysis. [file TPJ-103-1783-s012.docx]

**Table S3.** Primers used for vector construction and quantitative real time (qRT)-PCR analysis.

| **Accession No.** | | | **Primer set** | |
| --- | --- | --- | --- | --- |
|  |  |  | **Forward primer (5’-3’)** | **Reverse primer (5’ -3’)** |
| **For vector construction** | | | | |
| **CmBBX19-overexpression vector** | | | | |
| *CmBBX19*-OX | GTTCTAGAATGCGAACATTATGTGATGTTTGTG | | | TCCCCGGGTTTACTTGTCAGCTTCTCTTTTAAAGG |
| **CmBBX19-RNAi vector** | | | | |
| *CmBBX19*-Sense | TTGCTCGAGTTGAGCAACAAGATGGTCTTGGAG | | | TTATCGATAGAATCACTCGTAACGACATAGAAC |
| *CmBBX19*-Antisense | TGTCTAGATTGAGCAACAAGATGGTCTTGGAG | | | TTTGGTACCAGAATCACTCGTAACGACATAGAAC |
| **Subcellular localization** | | | | |
| CmBBX19 –Localization | CGTCTAGAATGCGAACATTATGTGATGTTTGTG | | | GTCGACGCTTGTCACCTTCTCTTTTAAAGG |
| **Transcriptional activation assay** | | | | |
| BD-CmBBX19 | TTGACGAGTACGGTGGGACCGGTATGCGAACATTATGTGA | | | AATGAAACCAGAGTTAAAGGCCTTTACTTGTCACCTTCTC |
| VP16 | CTTCTCAATAGAAATGCCCCCCCGACCGAT | | | CCCACCGTACTCGTCAATTC |
| **Yeast two-hybrid assay** | | | | |
| BD-CmBBX19 | GACGAATTCATGCGAACATTATGTGATGTTTGTG | | | GTCGACGCTTGTCACCTTCTCTTTTAAAGG |
| AD-CmABF3 | GACGAATTCATGAGTTCGTTTATCAACTTCA | | | GTCGACCCAAGGTCCAGTCAATGTTCTTC |
| AD-CmABF1 | GACCATGGAGATGAGTTCATTAATCAATCCCAAG | | | CAGGATCCCCAAGGTCCTGCTAACGTTCTT |
| AD-CmABF2a | GACGAATTCATGACTCTTGAGGAATTTTTGGT | | | GTCGACCCATGGACCAGAATTTGTCTTTC |
| AD-CmABF2b | GACGAATTCATGAACTACAAGAATTTTGGAAGTC | | | GTCGACCCATGGACCGGAAAGTGTCTTGCT |
| AD-CmABI5 | GACGAATTCATGGTTGTACCAGATTCAGAAAT | | | GTCGACAAACGGCACAACTCGAGGTTC |
| AD-CmABF3-C1 | Same as CmABF3 forward | | | GTCGACATCTATTGCAAAATTTGAGGTTG |
| AD-CmABF3-**△**C1 | GACGAATTCGGAAGTGTGTCGAATGATGG | | | Same as CmABF3 reverse |
| AD-CmABF3-C2C3 | Same as CmABF3-**△**C1 forward | | | GTCGACTAAACCACCTTCACCAAATGTAT |
| AD-CmABF3-bZIP | GACGAATTCCGAGGGAGGAAAAGCAGTGG | | | GTCGACCTGATTGTTATGCATATCCATC |
| AD-CmABF3-bZIPC4 | Same as CmABF3- bZIP forward | | | Same as CmABF3 reverse |
| BD-AtBBX19 | atggccatggaggccgaattcATGCGGATTTTGTGCGATG | | | atgcggccgctgcaggtcgacTCACTTCTCAGACTCTCGTTTAAAGG |
| AD-AtABF1 | gccatggaggccagtgaattcATGGGTACTCACATTGATATCAACAAC | | | acgattcatctgcagctcgagTCACCTTCTTACCACGGACCG |
| AD-AtABF2 | gccatggaggccagtgaattcATGGTAGTATGAATTTGGGGAATGA | | | acgattcatctgcagctcgagTCACCAAGGTCCCGACTCTG |
| AD-AtABF3 | gccatggaggccagtgaattcATGGGGTCTAGATTAAACTTCAAGAGC | | | acgattcatctgcagctcgagCTACCAGGGACCCGTCAATG |
| AD-AtABF4 | gccatggaggccagtgaattcATGGGAACTCACATCAATTTCAACA | | | acgattcatctgcagctcgagTCACCATGGTCCGGTTAATGTC |
| AD-AtABI5 | gccatggaggccagtgaattcATGGTAACTAGAGAAACGAAGTTGACG | | | acgattcatctgcagctcgagTTAGAGTGGACAACTCGGGTTCC |
| **Bimolecular fluorescence complementation (BiFC) assay** | | | | |
| CmBBX19- YFP^N^ | ccaaatcgactctagtctagaATGCGAACATTATGTGATGT | | | agcggtaccctcgaggtcgacTTACTTGTCACCTTCTCTTTT |
| CmABF3-YFP^C^ | gagaacacgggggactctagaATGAGTTCGTTTATCAACTTCAAGAAC | | | agcggtaccctcgaggtcgacCCAAGGTCCAGTCAATGTTCTTC |
| AtBBX19- YFP^N^ | gagaacacgggggactctagaATGCGGATTTTGTGCGATG | | | agcggtaccctcgaggtcgacCTTCTCAGACTCTCGTTTAAAGGGT |
| AtABF1-YFP^C^ | gagaacacgggggactctagaATGGGTACTCACATTGATATCAACAAC | | | agcggtaccctcgaggtcgacCCTTCTTACCACGGACCGG |
| AtABF2-YFP^C^ | gagaacacgggggactctagaATGGTAGTATGAATTTGGGGAATGA | | | agcggtaccctcgaggtcgacCCAAGGTCCCGACTCTGTCC |
| AtABF3-YFP^C^ | gagaacacgggggactctagaATGGGGTCTAGATTAAACTTCAAGAGC | | | agcggtaccctcgaggtcgacCCAGGGACCCGTCAATGTC |
| AtABF4-YFP^C^ | gagaacacgggggactctagaATGGGAACTCACATCAATTTCAACA | | | agcggtaccctcgaggtcgacCCATGGTCCGGTTAATGTCCT |
| AtABI5-YFP^C^ | gagaacacgggggactctagaATGGTAACTAGAGAAACGAAGTTGACG | | | agcggtaccctcgaggtcgacGAGTGGACAACTCGGGTTCCT |
| **Yeast one-hybrid** | | | | |
| pAbAi-proCmRAB18 | cttgaattcgagctcggtaccCTGAAATTAGACACGTACTTTTCA | | | agcacatgcctcgaggtcgacAACCGTACACGTGCAAGTTTGTA |
| AD-CmBBX19 | GACGAATTCATGCGAACATTATGTGATGTTTGTG | | | GTCGACGCTTGTCACCTTCTCTTTTAAAGG |
| **Electrophoretic mobility shift assay (EMSA)** | | | | |
| proRAB18 -probe | Biotin—GAAATTAGACACGTACTTTTCAGTGATAACATAAACATACTTACGTGTTC | | | Biotin—GAACACGTAAGTATGTTTATGTTATCACTGAAAAGTACGTGTCTAATTTC |
| proRAB18-coldprobe | GAAATTAGACACGTACTTTTCAGTGATAACATAAACATACTTACGTGTTC | | | GAACACGTAAGTATGTTTATGTTATCACTGAAAAGTACGTGTCTAATTTC |
| pGEX-CmABF3 | gatctggttccgcgtggatccATGAGTTCGTTTATCAACTTC | | | ctcgagtcgacccgggaattcTCACCAAGGTCCAGTCAATG |
| pGEX-CmBBX19 | gatctggttccgcgtggatccATGCGAACATTATGTGATGT | | | ctcgagtcgacccgggaattcTTACTTGTCACCTTCTCTTTT |
| **Dual-luciferase reporter assay vector** | | | | |
| SK- CmABF3 | GACGAATTCATGAGTTCGTTTATCAACTTC | | | GTCGACTCACCAAGGTCCAGTCAATG |
| SK- Cm BBX19 | CGTCTAGAATGCGAACATTATGTGATGTTTGTG | | | CAGGTACCTTACTTGTCACCTTCTCTTTTAAAGG |
| LUC-proCmRAB18 | gtcgacggtatcgataagcttCAAGAGAATGAACAAGATGCG | | | tgtttttggcgtcttccatggTCTTAAGTGAAAAAGATTTATTTGAG |
| **MIR VIGS vector** | | | | |
| I-miR-CmABF3-s | | gaTATCATTCGACACAGTTCCATtctctcttttgtattcc | | |
| II miR-CmABF3-a | | gaATGGAACTGTGTCGAATGATAtcaaagagaatcaatga | | |
| III miR-CmABF3*s | | gaATAGAACTGTGTCCAATGATTtcacaggtcgtgatatg | | |
| IV miR-CmABF3*a | | gaAATCATTGGACACAGTTCTATtctacatatatattcct | | |
| **For qRT-PCR analysis** | | | | |
| CmBBX19 | ATGATGGTGCTGGCAGGATG | | | TGACCATGCATCCGTTGAGG |
| CmUbiquitin | CTAATGAATGCTTACTGTGACCGAC | | | AGGCGAATCATCAGTACCAAGTG |
| CmRAB18 | ATGGTAGCGAACAGCACACA | | | CTGGTGACCTCCTCCTGGTA |
| CmRD29B | ACTCTTTTGATCGCCACTTGGA | | | GGCGATGTGAGTGCGGATAA |
| CmERD7 | AGATGCGTTGCGTGTTATATGTG | | | AGCGAGAACACAACAAAGGATAGA |
| CmLTI65 | ACATGAAGTTGGTTGTGGTGATG | | | TTGCAGTCGACATCTGCTAGTAT |
| CmABF3 | ACGAAGAACATTGACTGGACCT | | | ACATGCACACAGACGCATTT |
| CmABI1 | CGCTGCTTGTGCTGCTAGAT | | | CAGTGACGGGTTATGGGACG |
| CmDREB2 | GTCCCAAGCCACAACCTCTT | | | AACGCGTAAAGCTCCTGCTA |
| CmDREB5 | CACGCAGATAAAACACCGCC | | | CCAAAACATGCAACCGACCA |
| CmBBX22a | ACTGTGCCGGAAATGTGATCT | | | CTGGAACATTCCTTGCTGGC |
| CmBBX22b | AATACAACAACCCCGTGCCA | | | ACGCCAGTAAGCCCAAGAAA |
| CmBBX22c | TTGAGCGATCTCAACCAGGG | | | AACAGTCCGAGTCTCCCATC |
| CmBBX24 | CTGTGTAGAGGACAGAGCCCT | | | TTTTCGGGCTCTTGGGTGG |
| CmABF1 | TGCATACGGTCAAGGTGGTG | | | TGCTTTTTCTGCAACTCGTGG |
| CmABF2a | CCTTGGTGTTGTGGGGTTGA | | | AGAAAGGCGGAGATGATGGAA |
| CmABF2b | GCCTGTTTACGAGCCCTTGA | | | CGGACGGGTTAGCAAAGAGT |
| CmABI5 | CCTTTGGCAATCAAACGGCA | | | ACTCAAACAAGCATTGGCCG |
| CmABA2 | ACCACATCATCACCTGAATACCC | | | GGATTTGTTGCGGGAGGATG |
| CmPYL | CGAGACACCCGTTTTCACCA | | | GCAGTTTCCTCCCTATTGTGT |
| CmOST1 | GCGCACGTTTCATAACAGCA | | | AGGACCTAGAAAGTGATCCAGA |
| CmABI4 | TTCCTCGTCCACCATTTACCACC | | | AGGTGAGAGTGTGTTGGTTGTG |
| CmNYC1 | TCCACACAGCCTTATCGACT | | | TGCATCACCGGGCACTTAAA |
| CmSAG29 | GCTCCGACTTCAATGGTGTG | | | TGGAATCACCAAGAATCCCAGAA |
| CmABI5 | CCTTTGGCAATCAAACGGCA | | | ACTCAAACAAGCATTGGCCG |
| CmCXXS1 | CAGGATTTGCTCCAACTAGCC | | | TCGAAGAGTTGGCCTCAGAA |
| CmGRXC11 | AGACAACGCTGTGATCGTGT | | | GGGAAACTGCACTCTGGGTT |
| CmPRX12 | GCGGCAAAGGTGAAATTCGT | | | ACTCGAGGTAGGACTGCTCA |
| CmCSLG2 | CCTCCCGTCCAGCGAAATAA | | | GGGGTGTTCGGGGACTTTAG |
