# Supplementary material for: A zinc finger protein BBX19 interacts with ABF3 to affect drought tolerance negatively in chrysanthemum
Source: Plant J. 2020 Jul 21;103(5):1783–95. doi: 10.1111/tpj.14863 (PMC7496117; doi:10.1111/tpj.14863)
Supplement: Supplementary file 13 — Methods S1. Sequence analysis. [file TPJ-103-1783-s013.docx]

**Methods S1**

**Sequence analysis**

Alignments of the deduced amino acid sequence of CmBBX19 with the *Arabidopsis thaliana* BBX19 sequence, and of the deduced amino acid sequence of Cm ABF/AREB proteins with *A. thaliana* CmABF/AREB sequences were performed using BioEdit (http://www.mbio.ncsu.edu/BioEdit/bioedit.html) and ClustalW (http://www.ch.embnet.org/software/ClustalW.html). Phylogenetic analyses were performed using MEGA version 5 and the neighbor-joining method, with 1,000 bootstrap replicates.

AtBBX18 (AT2G21320) and AtBBX19 (AT4G38960) sequences were obtained from the TAIR database (www.arabidopsis.org) and PeBBX19 (XP_011047357), VvBBX19 (XP_002267957), BvBBX19L (XP_010690759), SlBBX19L (XP_004230952) and OsSTOL (XP_006660878) sequences were obtained from NCBI (http://www.ncbi.nlm.nih.gov/). *A. thaliana* BBX family sequences were obtained from the TAIR database: AtBBX18 (AT2G21320), AtBBX19 (AT4G38960), AtBBX20 (AT4G39070). AtBBX21 (AT1G75540), AtBBX22 (AT1G78600), AtBBX23 (AT4G10240), AtBBX24 (AT1G06040), AtBBX25 (AT2G31380). *A. thaliana* ABF/AREB sequences were obtained from the TAIR database: ABF1 (AT1G49720), ABF2 (AT1G45249), ABF3 (AT4G34000), ABF4 (AT3G19290) and ABI5 (AT2G36270).

**Western blotting analysis**

The top fifth and sixth fully expanded chrysanthemum leaves from 60-day-old *CmBBX19*-OX lines and wild-type plants were used for western blotting analysis. Protein extraction was performed with 4×SDS buffer (0.2 M Tris, 0.4 M DTT, 8% [W/V] SDS, 0.4% [W/V] Bromophenol blue, 40% [V/V] Glycerol), and the protein extracts were separated on 10% SDS-PAGE gels, and transferred to a NC membrane (Millipore). Anti-GFP (1:5000 v/v) (Abmart; catalog no. P30010M), and anti-Actin (1:10000 v/v) (Bioeasytech; catalog no. A0480) were used as primary antibodies. And anti-rabbit horseradish peroxidase-conjugated secondary antibody was used as the secondary antibody. Protein signals were detected using the LAS-3000 imaging system (Fuji Film) with ECL solution (Millipore).
